# A small molecule inhibitor of mutant IDH2 rescues cardiomyopathy in a D-2-hydroxyglutaric aciduria type II mouse model

**DOI:** 10.1007/s10545-016-9960-y

**Published:** 2016-07-28

**Authors:** Fang Wang, Jeremy Travins, Zhizhong Lin, Yaguang Si, Yue Chen, Josh Powe, Stuart Murray, Dongwei Zhu, Erin Artin, Stefan Gross, Stephanie Santiago, Mya Steadman, Andrew Kernytsky, Kimberly Straley, Chenming Lu, Ana Pop, Eduard A. Struys, Erwin E. W. Jansen, Gajja S. Salomons, Muriel D. David, Cyril Quivoron, Virginie Penard-Lacronique, Karen S. Regan, Wei Liu, Lenny Dang, Hua Yang, Lee Silverman, Samuel Agresta, Marion Dorsch, Scott Biller, Katharine Yen, Yong Cang, Shin-San Michael Su, Shengfang Jin

**Affiliations:** 1 Agios Pharmaceuticals Inc., 88 Sidney Street, Cambridge, MA 02139-4169 USA; 2 Oncology Business Unit, WuXi AppTec, Shanghai, China; 3 Metabolic Unit, Department of Clinical Chemistry, VU University Medical Center/ Neuroscience Campus, Amsterdam, The Netherlands; 4 Institut National de la Santé et de la Recherche Médicale, INSERM U1170, Villejuif, France; 5 Institut Gustave Roussy, Villejuif, France; 6 Regan Pathology/Toxicology Services, Ashland, OH USA

## Abstract

D-2-hydroxyglutaric aciduria (D2HGA) type II is a rare neurometabolic disorder caused by germline gain-of-function mutations in isocitrate dehydrogenase 2 (*IDH2*), resulting in accumulation of D-2-hydroxyglutarate (D2HG). Patients exhibit a wide spectrum of symptoms including cardiomyopathy, epilepsy, developmental delay and limited life span. Currently, there are no effective therapeutic interventions. We generated a D2HGA type II mouse model by introducing the *Idh2R140Q* mutation at the native chromosomal locus. Idh2R140Q mice displayed significantly elevated 2HG levels and recapitulated multiple defects seen in patients. AGI-026, a potent, selective inhibitor of the human IDH2R140Q-mutant enzyme, suppressed 2HG production, rescued cardiomyopathy, and provided a survival benefit in Idh2R140Q mice; treatment withdrawal resulted in deterioration of cardiac function. We observed differential expression of multiple genes and metabolites that are associated with cardiomyopathy, which were largely reversed by AGI-026. These findings demonstrate the potential therapeutic benefit of an IDH2R140Q inhibitor in patients with D2HGA type II.

**Electronic supplementary material:**

The online version of this article (doi:10.1007/s10545-016-9960-y) contains supplementary material, which is available to authorized users.

## Introduction

D-2-hydroxyglutaric aciduria (D2HGA) is a rare, inborn, neurometabolic disorder with two genetically distinct types (Kranendijk et al 2012). Type I is caused by germline autosomal recessive, loss-of-function mutations in the mitochondrial D-2-hydroxyglutarate dehydrogenase gene (*D2HGDH*) (Kranendijk et al 2010a), impairing the enzyme’s ability to convert D-2-hydroxyglutarate to alpha-ketoglutarate (αKG) (Struys et al 2005). Type II results from autosomal dominant, gain-of-function mutations in the Arg^140^ residue of the mitochondrial isocitrate dehydrogenase 2 gene (*IDH2*), predominantly the IDH2R140Q mutation (Kranendijk et al 2010b). Somatic mutations in IDH2 and IDH1 (cytosolic isoform) have been found in multiple neoplasms (Mardis et al 2009; Yan et al 2009), and confer neomorphic activity, allowing the mutant enzyme to catalyze the reduction of αKG to 2-hydroxyglutarate (2HG) (Dang et al 2009; Gross et al 2010).

In D2HGA, *IDH2* and *D2HGDH* mutations both result in 2HG accumulation, accompanied by developmental delay, hypotonia and seizures, although there are phenotypic differences between the two types. Type II (*IDH2* mutation) is associated with earlier onset, more severe developmental delay, higher seizure frequency and reduced life expectancy than type I (*D2HGDH* mutation), and ∼50 % of patients have cardiomyopathy, which is not observed in type I (Kranendijk et al 2012). These variations appear to correlate with higher 2HG levels in type II disease (Kranendijk et al 2012). There are currently no effective therapeutic interventions other than supportive use of anticonvulsants to control seizures.

Cancer metabolism studies demonstrated that 2HG is an oncometabolite, promoting epigenetic aberrations via inhibition of αKG-dependent dioxygenases, including DNA and histone demethylases, and impaired cellular differentiation (Figueroa et al 2010; Chowdhury et al 2011; Xu et al 2011). We have developed a series of selective, potent inhibitors of the IDH2R140Q mutant protein, and demonstrated that inhibition of 2HG production with these compounds reverses epigenetic re-wiring and promotes normal cellular differentiation, with preliminary clinical data indicating clinical activity in advanced hematologic malignancies (Wang et al 2013a; Stein et al 2014; Kernytsky et al 2015). Due to the rarity of D2HGA type II (Kranendijk et al 2012), and lack of an optimal preclinical model system, understanding of disease pathophysiology is lacking. To address this knowledge gap, we developed and characterized a knock-in (KI) mouse model of D2HGA type II, which we used to evaluate the therapeutic potential of the IDH2R140Q inhibitor AGI-026, which was selected from our series due to its favorable potency, relative selectivity, and especially its ability to penetrate the blood-brain barrier.

## Materials and methods

### Mouse husbandry and breeding

Mice were bred and maintained in a barrier facility under pathogen-free conditions (WuXi AppTec, Shanghai, China). Mice were housed under 12/12 h light/dark cycles and given *ad libitum* access to food and water. Experiments were conducted in accordance with protocols approved by the Institutional Animal Care and Use Committee. Prior to efficacy studies, a tolerability study was performed in which wild type (wt) mice were administered 50 mg/kg AGI-026 for 7 days. We observed no changes in body weight, animal alertness or activity, eating or general behavior, no gross phenotypic changes in bone marrow cells upon flow cytometry, and no histopathological changes in liver, spleen, brain and heart. For efficacy assessment, AGI-026 (2 or 10 mg/kg) or vehicle (0.5 % *w*/*v* methylcellulose/0.25 % tween-80 diluted with purified water) was administered to male and female mice by oral gavage, with initiation at 4–5 weeks old (post-weaning). Body weight and overall health were continuously monitored.

### Generation of D2HGA type II mouse model

The targeting vector was assembled as shown in Supplementary Fig. 1a and contained a loxP-flanked STOP (LSL) cassette in the preceding intron for conditional expression of the Idh2R140Q protein in a Cre-recombinase-dependent manner, and genomic fragments of the *Idh2* gene including a 3.0 kb 5′ arm and 7.6 kb 3′ arm retrieved from 129S6/SvEv BAC DNA (Source BioScience, UK) via an E. coli-based chromosome engineering system (Lee et al 2001). The 3′ arm contained the murine *Idh2* exon 4 bearing the R140Q mutation, which was created by PCR-based mutagenesis. An FRT-flanked neomycin resistance cassette (Neo^r^) and a thymidine kinase gene served as selectable markers for genomic integration. The linearized targeting vector was electroporated into 129S6/SvEv mouse embryonic stem (ES) cells. Following homologous recombination in ES cells, an expression vector of Flp-recombinase was transiently transfected to delete the Neo^r^ cassette. Targeted ES cells were identified by PCR and sequencing analyses and then injected into C57BL/6J blastocysts, which were transferred to pseudopregnant females to produce chimeric mice. Chimeric males from two ES cell clones gave germline transmission of the conditional KI IHD2 allele (IDH2^LSL^). The chimeric mice were bred to C57BL/6J mice for germline transmission. Mutant mice were crossed with CMV-Cre transgenic mice on a C57BL/6J background to generate heterozygous Idh2R140Q mice by deleting the STOP cassette. The presence of systemic Idh2R140Q mutation was confirmed by PCR analysis of tail genomic DNA using primers spanning the position of the residual loxP site (5′- CTGTGCCACTGAGCTACATCTCCAG-3′ and 5′- CGTTTTTACCAGGTCCACATGGAAG -3′), generating a 150-bp PCR product in Idh2wt mice and a 204-bp PCR product in KI mice. Mice were also analyzed for the presence of Idh2R140Q by sequencing using PCR primers (5′-CCTGAGCCGAATGCTTTTAAG-3′ and 5′- CTCACGCTCCTTACTGGAC-3′). Idh2wt littermates of Idh2R140Q mice served as controls for all analyses.

No formal sample size calculation was performed but sufficient numbers of mice were bred to ensure at least 20 animals per treatment group for the AGI-026 treatment studies; sufficient numbers were obtained to achieve statistical significance. Animals were selected for inclusion based on confirmation of Idh2R140Q/wt genotype by PCR and sequencing, and elevated plasma 2HG levels (see below for method); no other inclusion/exclusion criteria were applied. Mice were randomized using the “RAND” function in Microsoft Excel and assignment to treatment groups based on their randomization number (0–0.25 to group 1, 0.25–0.50 to group 2, 0.50–0.75 to group 3, and 0.75–1 to group 4). This was a non-blinded study. For each of the efficacy experiments, all animals were treated simultaneously to ensure consistency in experimental conditions and age of animals.

### 2HG assessment

Mice were euthanized by CO_2_ inhalation and blood was collected by retro-orbital sampling (biweekly assessments) or intracardiac puncture (end of study). Tissues including heart, brain, liver, and skeletal muscle were dissected, flash frozen in liquid nitrogen and stored at −80 °C until analysis. Bone marrow was flushed out from the tibia and femur in phosphate-buffered saline without Mg^2+^ or Ca^2+^, and the viable cell number determined by cell counting after trypan blue staining. Total 2HG levels were assessed as a surrogate marker for D2HG levels using liquid chromatography-tandem mass spectrometry (LC-MS) as previously described (Dang et al 2009). Plasma 2HG levels were assessed every 2 weeks during AGI-026 treatment.

### Whole body histopathology

At necropsy, mouse tissues were trimmed and fixed in 10 % neutral buffered formalin. Fixed tissues were processed, paraffin-embedded, sectioned, and stained with hematoxylin and eosin according to standard laboratory protocols. Histopathological evaluation was conducted by a veterinary pathologist.

### Echocardiography

Mice were anesthetized with pentobarbital sodium (1 mg/kg) and immobilized on a heating platform, ventral side up, to maintain body temperature at 37.0 ± 0.5 °C. Heart rate and respiratory physiology were continuously monitored during examination. M mode echocardiographic studies for determination of cardiac left ventricular hypertrophy (left ventricular mass and ejection fraction) were performed using a Vevo® 2100 system (VisualSonics, Toronto, Canada) with a 30 MHz transducer.

### Biochemical and cellular properties of AGI-026

AGI-026 was prepared as a 10 mM stock in dimethyl sulfoxide (DMSO) and diluted to 50× final concentration in DMSO. IDH2 mutant homodimer enzyme activity and IDH2wt/IDH2 mutant heterodimer enzyme activity of converting αKG to 2HG was measured in an end-point assay of NADPH depletion. In this assay, the remaining cofactor was measured at the end of the reaction period by the addition of a catalytic excess of diaphorase and resazurin to generate a fluorescent signal in proportion to the amount of NADPH remaining. IDH2wt homodimer enzyme activity of converting isocitrate to αKG was measured in a continuous assay directly coupling NADPH production to conversion of resazurin to resorufin by diaphorase. In both cases, resorufin was measured via fluorescence (λ_ex_ = 544 nm, λ_em_ = 590 nm).

Specifically, the IDH2R140Q homodimer enzyme was diluted to 0.31 μg/mL in 40 μL assay buffer (150 mM NaCl, 50 mM K_2_HPO_4_ pH 7.5, 10 mM MgCl_2_, 10 % glycerol, 0.05 % BSA) containing 1 μL of 50× compound dilution series, 5 μM NADPH cofactor, and 2.5 mM β-mercaptoethanol, and the mixture incubated at 25 °C for 16 h. The reaction was then initiated with the addition of 10 μL of 8 mM αKG in assay buffer. The reactions were run for 40 min at room temperature and terminated by the addition of 25 μL of detection buffer (36 μg/mL diaphorase, 18 μM resazurin, in 1× assay buffer), shaken for 1 min on a laboratory shaker, and read on a SpectraMax 384-well plate reader as described above. The IDH2wt homodimer enzyme was diluted to 0.1 μg/mL in 40 μL assay buffer containing 1 μL of 50× compound dilution series, 35 μM NADP+ cofactor, and 2.5 mM β-mercaptoethanol, and the mixture incubated at 25 °C for 16 h. The reaction was then initiated with the addition of 10 μL of a substrate mix containing 0.2 mM isocitrate, 60 μg/mL diaphorase, and 200 μM resazurin in assay buffer. The reactions were run at 25 °C for 30 min, stopped by the addition of 25 μL of 6 % SDS solution, and read on a SpectraMax 384-well plate reader. The IDH2wt/R140Q heterodimer enzyme was diluted to 0.25 μg/mL in 40 μL assay buffer containing 1 μL of 50× compound dilution series, 42.5 μM NADP+/5 μM NADPH cofactor mix, and 2.5 mM β-mercaptoethanol. This mixture was incubated at 25°C for 16 h. The reaction was then initiated with the addition of 10 μL of 6.25 mM αKG in assay buffer. The reactions were run for 50 min at room temperature and terminated by the addition of 25 μL of detection buffer, shaken for 1 min on a laboratory shaker, and read on a SpectraMax 384-well plate reader.

Cellular potency (IC_50_ determined using levels of the enzyme product 2HG in culture medium) was tested in mouse ES Idh2R140Q cells and the U87 glioblastoma cell line ectopically overexpressing IDH2R140Q, as previously described (Wang et al 2013a). Selectivity of AGI-026 was determined using a panel of dehydrogenases (Wang et al 2013a) and an enzyme panel screen from CEREP (Poitiers, France). 2HG in culture medium was measured by LC-MS. Total 2HG level was measured as a surrogate for D2HG.

Lymphoblast cells were obtained from three unrelated patients with 2DHGA type II and cultured as previously described (Wickenhagen et al 2009). Cells were seeded in a 96-well plate at 50,000 cells per well in 200 μl of complete medium. Duplicate wells were seeded to obtain cell counts at 48 h post-culture (no compound administered), and indicated 150,000 cells per well. Cells were incubated with varying concentrations of AGI-026 and culture medium was collected after 48, 72, and 96 h. A 10 mM stock was made in DMSO and diluted in culture medium, with a 10X dilution range made from 10 μM down to 10 pM (seven concentrations by tenfold sequential dilution). D2HG and L2HG in culture medium were measured by LC-MS as described previously (Struys et al 2004).

### 5hmC and 5mC assessment

Genomic DNA was extracted from approximately 25 mg of heart tissue using the QIAamp DNA Mini Kit (Qiagen) according to the manufacturer’s instructions. For each sample, 1 μg of DNA was degraded into individual nucleosides using DNA Degradase Plus™ (Zymo Research Corporation, Irvine, CA). Individual nucleosides were measured using a high-performance liquid chromatography-tandem mass spectrometry (HPLC-MS/MS) system consisting of a Dionex UltiMate® 3000 UHPLC system (Thermo Scientific, Sunnyvale, CA) containing a Kinetex HILIC column (2.1 × 100 mm, 1.7 μm; Phenomenex Inc., Torrance, CA) connected to a TSQ Vantage mass spectrometer (Thermo Scientific). Quantification was performed using area-based linear regression curves derived from calibration standards containing internal standard solutions. 5mC and 5hmC levels were calculated as a concentration percentage ratio of total deoxycytidine species; %5mC = 5-methyl-2′-deoxycytidine/(5-methyl-2′-deoxycytidine + 5-hydroxymethyl-2′-deoxycytidine + 2′-deoxycytidine) and %5hmC = 5-hydroxymethyl-2′-deoxycytidine/(5-methyl-2′-deoxycytidine + 5-hydroxymethyl-2′-deoxycytidine + 2′-deoxycytidine).

### RNA sequencing

Total RNA from frozen tissues was isolated using the RNeasy Mini Kit (Qiagen, Germany) according to the manufacturer’s instructions. RNA was tested for quality using a spectrophotometer, and was only accepted if the RNA Integrity Number (RIN) was greater than 9.0. RNA was extracted and sequenced on a HiSeq 2000 system (Illumina Inc., San Diego, CA) with paired-end 100 base pair reads. RNA-Seq reads were aligned to the mouse reference genome (NCBI build 37.2) with TopHat 2 (Trapnell et al 2009; Kim et al 2013); transcripts were assembled and abundance was estimated with Cufflinks (Trapnell et al 2010); differential expression was assessed with CuffDiff (Trapnell et al 2013). Most conditions had three animals per cohort, allowing for robust estimation of the significance of expression changes with respect to animal-to-animal variation (q-value <0.05).

### Quantitative PCR

RNA was extracted from mouse heart tissue using the RNeasy Mini Kit and quantitative PCR carried out using TaqMan Universal Master Mix II with UNG (Applied Biosystems) on a 7900 HT Real-Time PCR Detection System (Applied Biosystems) according to standard protocols, as previously described (Wang et al 2013a). PCR primers were murine Six1 (Mm00808212_ml, Life Technologies), murine Nmrk2 (Mm01172899_g1, Life Technologies), and endogenous control 18S (4333760, Life Technologies).

### Gene set enrichment analysis

A functional analysis of differentially expressed genes was performed using the DAVID database (Dennis et al 2003; Huang et al 2009).

### Text mining

We used the I2E indexing/semantic search tool (Linguamatics, Cambridge, UK) for sentence identification, part of speech tagging, and semantic analysis of sentences according to its embedded linguistic rules (Hale 2005). Target genes and relational identification were correctly identified and parsed by NLP algorithms within the I2E tool. Extracted relational data was curated to disambiguate potential false positive hits (Chen et al 2005).

### Heart tissue metabolite profiling

Hearts that had been snap-frozen by immersion in liquid nitrogen immediately after dissection (maximum time of 5 minutes between sacrifice and freezing) were cut into pieces on dry ice, homogenized (TissueLyser II, Qiagen), and extracted with 80/20 MeOH/H_2_O (*v/v*) at a ratio of 150 μL extraction solution to 5 mg tissue. Homogenized samples were centrifuged at 14,000 rpm for 15 min at 4°C. A volume of supernatant equivalent to 1 mg of tissue per well (30 μL) was transferred to 96-well plates and evaporated under reduced pressure. Samples were analyzed using LC-MS. Prior to injection, dried extracts were reconstituted (30 μL) in LC-MS-grade water. Relative abundance of metabolites was evaluated by high resolution accurate mass detection (HRAM). HRAM data was acquired using a Q Exactive™ Orbitrap mass spectrometer (Thermo Scientific) operated in both positive and negative ion modes. LC-MS in positive mode was achieved as previously described (Jha et al 2015). LC-MS in negative ion mode was achieved using an Acquity UPLC® T3 C_18_ (3 μm, 2.0 × 150 mm) column (Waters Corporation, Milford, MA) and by implementation of a gradient elution program as previously described (Buescher et al 2010). Data were normalized by weight.

### Statistical analysis

Statistical analyses were conducted using GraphPad Prism (GraphPad Software Inc., La Jolla, CA).

## Results

### Idh2R140Q KI mice display a D2HGA type II phenotype

Constitutive expression of the Idh2R140Q mutant protein was achieved by introducing the R140Q mutation into the native *Idh2* locus using a targeting vector, and systemic heterozygous *Idh2R140Q* mutation confirmed by PCR genotyping and sequencing (Supplementary Fig. 1a-c). Idh2 wild type (Idh2wt) littermates served as controls for all analyses.

Idh2R140Q mice were viable and fertile, but obtained at a slightly lower than expected Mendelian ratio (Supplementary Fig. 2a), suggesting subtle prenatal abnormality. Substantially higher mortality was observed in Idh2R140Q versus Idh2wt mice (50 vs. 6.5 %) within 1 week of birth. Idh2R140Q mice surviving after 1 week had lower body weight than Idh2wt littermates during the first 5 weeks of life (Supplementary Fig. 2b); this characteristic gradually disappeared after week 5.

**Fig. 1:**
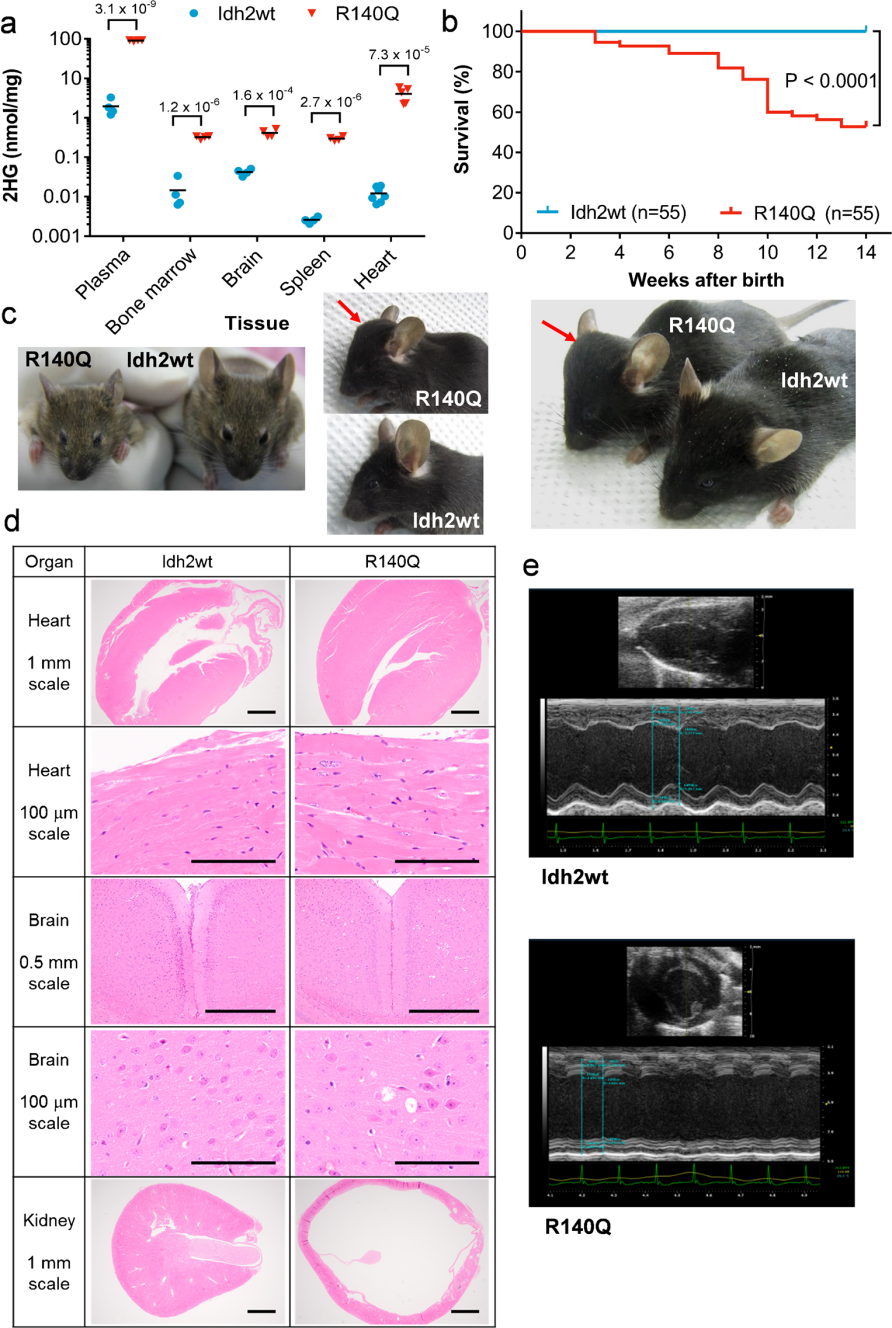
Characteristics of Idh2R140Q KI mice. (**a**) 2HG levels in plasma (nmol/mL), bone marrow, brain, spleen (all *n* = 4), and heart (*n* = 7 for Idh2wt and *n* = 5 for Idh2R140Q) in Idh2R140Q and Idh2wt mice. *Black horizontal bars* indicate mean. Statistical significance (p) calculated with t tests using the Holm-Sidak method (p values shown above brackets). (**b**) Kaplan-Meier survival curves in Idh2R140Q and Idh2wt mice from week 3. Statistical significance (p) tested using the log-rank (Mantel-Cox) test. (**c**) Abnormal phenotypes (*red arrow*) in Idh2R140Q mice: runting, facial dysmorphism, and abnormal shape of the head. (**d**) Histology of the heart, brain, and kidney in Idh2R140Q mice at approximately 12 weeks of age (hematoxylin and eosin stained). Longitudinal sections of the whole heart demonstrate ventricle wall thickening, and cardiomyocytes show hypertrophy. Transverse sections of the mid-brain showed vacuoles were consistently present in the lateral septal nuclei, various strata of the CA1 and CA3 areas of the hippocampus, the dentate gyrus, and the anterior cingulate and retrosplenial cortices, but were also observed laterally and ventrally in the cerebrum to involve the motor (shown), somatosensory and piriform cortices, particularly in the deeper neuronal layers (3 − 6), and were also sometimes present in the amygdala and medulla. Transverse sections of the whole kidney illustrate hydronephrosis, showing thinning of the cortex and almost total loss of the medulla. Histology is representative of six mice per group. (**e**) Echocardiographs demonstrating cardiac functional defects in 12-week old Idh2R140Q mice

Analysis of plasma, bone marrow, brain, spleen, and heart demonstrated dramatically higher 2HG levels in Idh2R140Q mice than Idh2wt littermates (Fig. 1a). Heart tissue displayed the highest 2HG levels, correlating with observations of the highest wild-type Idh2 mRNA and protein levels in heart tissue. By week 14, mortality reached 50 % in Idh2R140Q mice, whereas all Idh2wt littermates survived (Fig. 1b); most deaths were sudden. Periodic seizures occurred in 25 % of Idh2R140Q mice and 10 % displayed runting or abnormalities of the face and head (Fig. 1c), consistent with clinical reports of patients with D2HGA (Kranendijk et al 2012).

### Idh2R140Q KI mice exhibit histopathological abnormalities

Whole-body histopathology in Idh2R140Q mice identified abnormalities of the cardiovascular, central nervous and urinary systems. Compared with Idh2wt littermates, Idh2R140Q mice had enlarged hearts, increased left ventricular mass/body weight ratio (LVM/BW) and lower ejection fraction (EF) (Supplementary Table 1), apparent as early as week 4. Histopathology indicated increased ventricular wall thickness, decreased luminal space, and hypertrophy of cardiomyocytes characterized by cytoplasmic and nuclear enlargement (Fig. 1d). On echocardiography, Idh2R140Q mice exhibited severely decreased left ventricular wall motion and increased dilation without a change in heart rate (Fig. 1e). These data indicate cardiomyopathy, consistent with human D2HGA, and may contribute to the shortened life span and sudden deaths observed.

Developmental delay and seizures are observed in patients with D2HGA (Kranendijk et al 2012). In Idh2R140Q mice, vacuoles were consistently present in numerous brain structures (Fig. 1d), although their pathological significance in this model is not yet clear (Supplementary material).

Severe hydronephrosis was evident, with massive dilation of the renal pelvis and thinning of the renal cortex (Fig. 1d), and increased kidney weight, blood urea nitrogen and creatinine values (Supplementary Table 1), suggesting functional renal obstruction. This was surprising, as renal abnormalities have not been described in patients with D2HGA.

Overall, the Idh2R140Q KI mouse model recapitulates cardiomyopathy and other disease features observed in patients with D2HGA type II, thus providing a tool to further elucidate the disease mechanism and evaluate treatment approaches. Further analyses reported here focus mainly on cardiac pathology.

### AGI-026 inhibits 2HG production in IDH2R140Q mutant cells

**Fig. 2:**
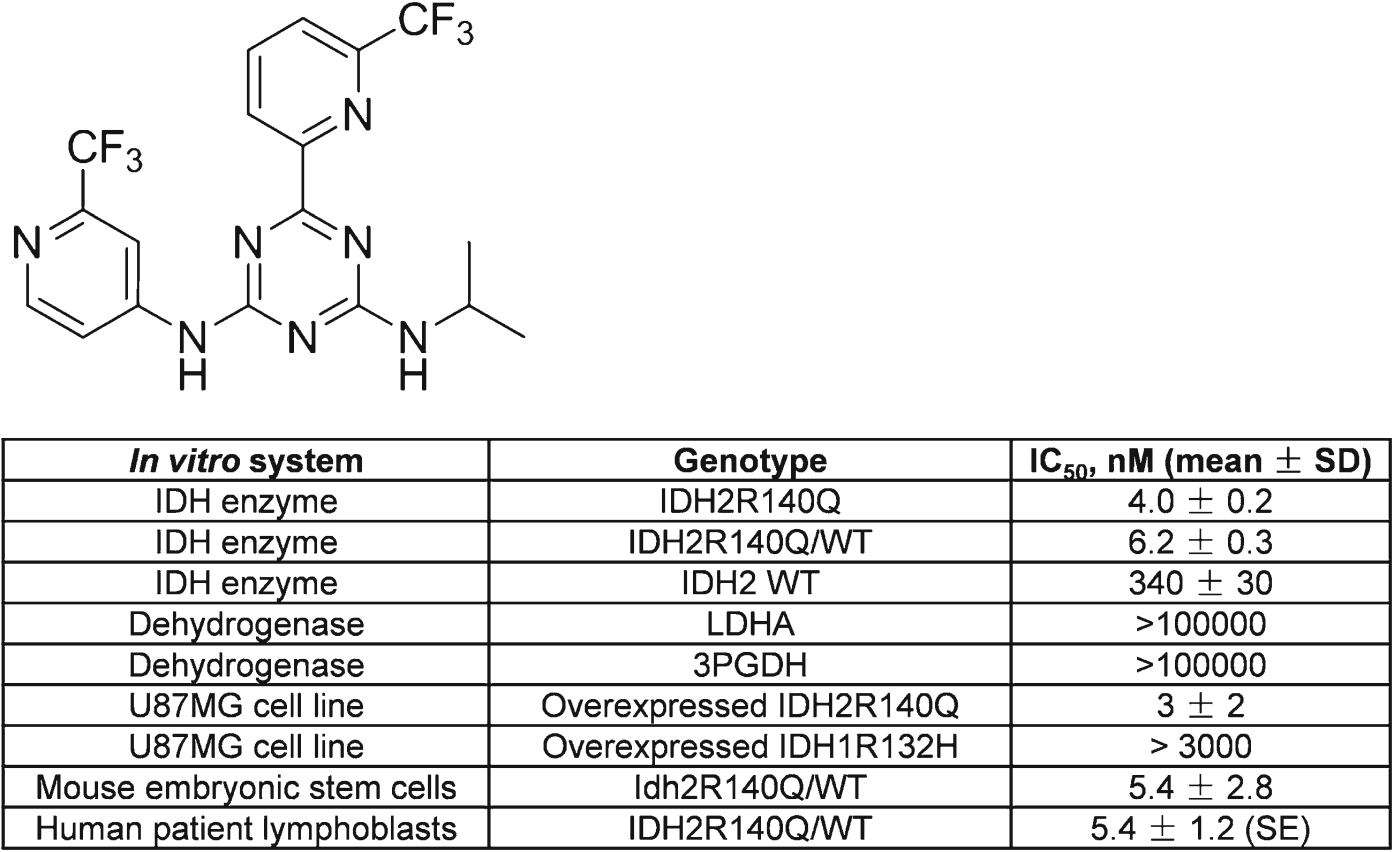
AGI-026, a brain-penetrant, potent and selective IDH2R140Q mutant inhibitor. Structure and biochemical properties of AGI-026. IC_50_ for 2HG production was measured *in vitro* for IDH2R140Q homodimer and IDH2wt/R140Q heterodimer recombinant purified enzymes. IC_50_ for αKG production was measured *in vitro* for IDH2wt homodimer recombinant purified enzyme. IC_50_ for 2HG production was measured in: the engineered glioblastoma U87MG cell line overexpressing IDH2R140Q or IDH1R132H; mouse Idh2R140Q embryonic stem cells; lymphoblast cells obtained from three unrelated patients with D2HGA type II. Mouse Idh2R140Q and human IDH2R140Q show 95.13 % identity

The triazine small molecule, AGI-026, is an orally bioavailable, selective, potent inhibitor of the human IDH2R140Q-mutant enzyme (IC_50_ = 4.0 ± 0.2 nM [mean ± SD]; Fig. 2) (International patent application WO2013102431A1). It did not exhibit inhibition in a panel of dehydrogenases, including lactate dehydrogenase and 3-phosphoglycerate dehydrogenases. The IC_50_ of 2HG production was similar in cultured lymphoblasts from patients with D2HGA type II (5.4 ± 1.2 nM [mean ± SE]) and Idh2R140Q KI mouse embryonic stem cells (5.4 ± 2.8 nM [mean ± SD]). AGI-026 displayed excellent penetration of the blood-brain barrier in mice after a single oral dose of 50 mg/kg (at 12 h post-dose, total brain to plasma concentration ratio = 1.6 in Idh2wt and 1.9 in Idh2R140Q mice).

### AGI-026 rescues the 2DHGA type II phenotype in Idh2R140Q mice

**Fig. 3:**
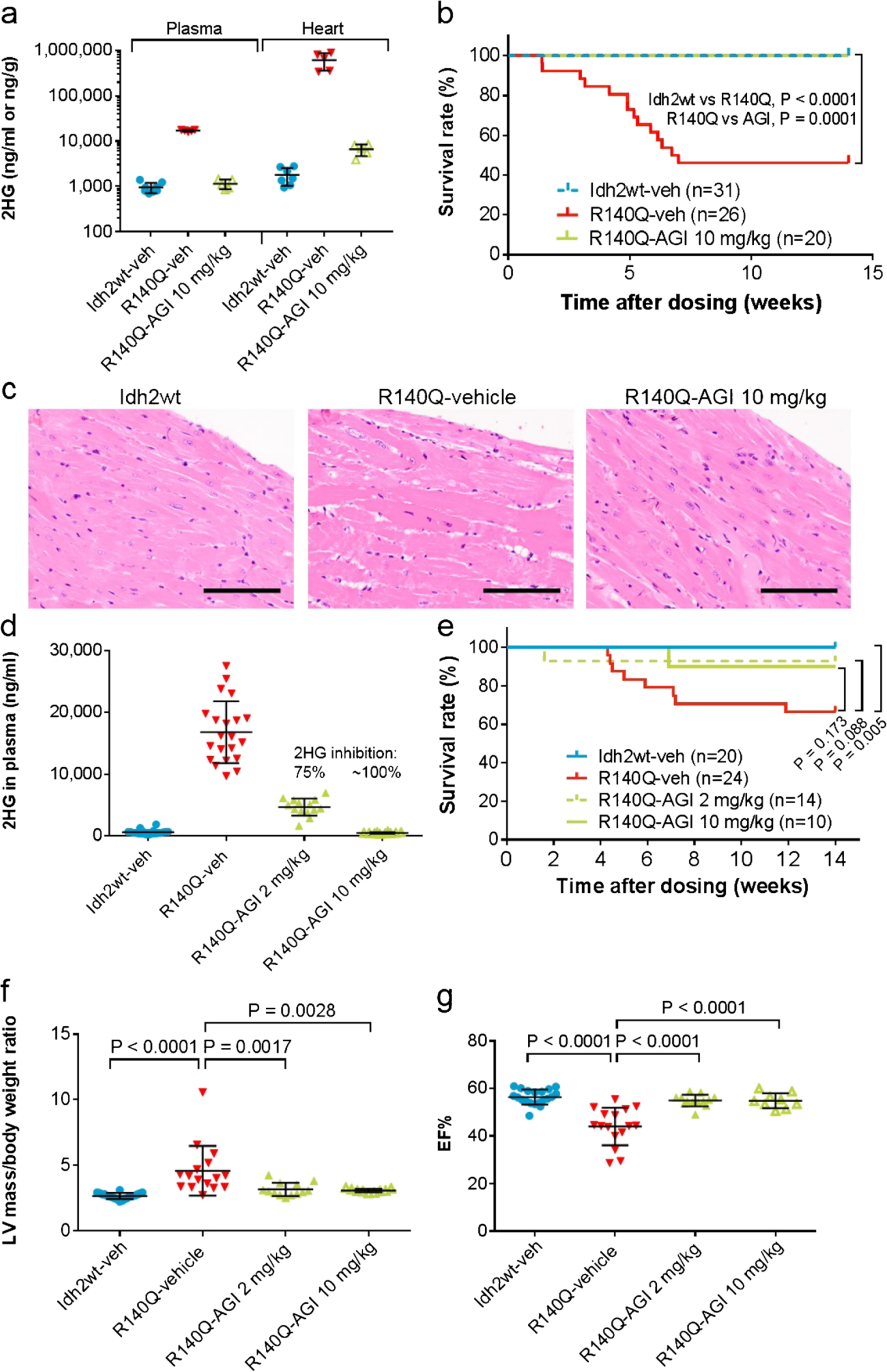
Efficacy of AGI-026 in reversing the phenotype observed in Idh2R140Q mice. Treatment was administered twice daily for 13 weeks. (**a**) Inhibition of 2HG in plasma and heart following 6 weeks’ treatment with AGI-026 10 mg/kg compared with vehicle (*n* = 8 for plasma, *n* = 7 for heart Idh2wt-veh, and *n* = 5 for other groups). (**b**) Kaplan-Meier survival curves in Idh2R140Q mice treated with 10 mg/kg AGI-026 or vehicle and Idh2wt mice receiving vehicle. Statistical significance (p) tested using the log-rank (Mantel-Cox) test. (**c**) Histopathological analysis of heart tissue in Idh2wt mice and Idh2R140Q mice with and without AGI-026 treatment, showing rescue of cardiac hypertrophy (cytoplasmic and nuclear enlargement) with treatment (scale bar denotes 100 μm). (**d**) Inhibition of 2HG in plasma following 6 weeks’ treatment with AGI-026 compared with vehicle in the second efficacy study (*n* = 20 for Idh2wt-veh, *n* = 21 for Idh2R140Q-veh, *n* = 14 for Idh2R140Q-AGI 2 mg/kg, and *n* = 10 for Idh2R140Q-AGI 10 mg/kg); % inhibition calculated as 100 % in Idh2wt-veh and 0 % in Idh2R140Q-veh. (**e**) Kaplan-Meier survival curves in Idh2R140Q mice treated with 2 mg/kg AGI-026, 10 mg/kg AGI-026 or vehicle and Idh2wt mice receiving vehicle. Statistical significance (p) tested using the log-rank (Mantel-Cox) test. (**f**) Left ventricular mass/body weight ratio and (**g**) ejection fraction assessed by echocardiogram at week 13 (*n* = 20 for Idh2wt-veh, *n* = 16 for Idh2R140Q-veh, *n* = 13 for Idh2R140Q-AGI 2 mg/kg, and *n* = 9 for Idh2R140Q-AGI 10 mg/kg). Error bars represent mean ± standard deviation; statistical significance (p) was tested with one-way ANOVA with Sidak’s multiple comparison test

To assess the efficacy of AGI-026 in rescuing the pathophysiological defects in our mouse model, AGI-026 10 mg/kg or vehicle was given by oral gavage twice daily (BID) for 13 weeks to Idh2R140Q mice, with dosing initiated at 4–5 weeks of age. AGI-026 was well tolerated: body weight was maintained and mice were clinically indistinguishable from Idh2wt controls. AGI-026 achieved 99 % reduction in 2HG in both plasma and heart tissue (Fig. 3a). The survival rate was 100 % in Idh2R140Q mice treated with AGI-026 (Idh2R140Q-AGI) but only 46 % in Idh2R140Q mice receiving vehicle (Idh2R140Q-veh) (Fig. 3b). Administration of a 50 mg/kg dose did not provide additional 2HG reduction or survival benefit (data not shown). At study end, three females and three males were randomly selected from each group for histopathological examination. Compared with Idh2R140Q-veh mice, Idh2R140Q-AGI mice had a lower incidence of myocardial hypertrophy and sub-gross cardiomegaly (Fig. 3c; Supplementary Table 2) and a lower average number of brain sites affected by vacuolation (Supplementary Table 3). Administration of AGI-026 had no effect on the incidence or severity of hydronephrosis (data not shown).

Given that we observed 100 % survival and reduced incidence of cardiac abnormalities at 10 mg/kg, we performed a second study with a lower dose. Following administration of AGI-026 at 2 mg/kg and 10 mg/kg BID continuously for 13 weeks, plasma 2HG was inhibited by 75 % and ∼100 %, respectively (Fig. 3d); the survival rate was 93 and 90 % in Idh2R140Q-AGI mice receiving 2 mg/kg and 10 mg/kg, respectively, and 67 % in Idh2R140Q-veh mice (Fig. 3e). Echocardiography on all surviving mice at week 13 showed significantly lower LVM/BW and significantly higher EF in Idh2R140Q-AGI versus Idh2R140Q-veh mice (Fig. 3f, g), indicating prevention of deterioration in cardiac function by AGI-026. No differences were observed between the two AGI-026 doses, indicating that doses achieving ≥75 % reduction of plasma 2HG are sufficient to prevent cardiac hypertrophy in this model.

### Continuous administration of AGI-026 is required to sustain benefit

**Fig. 4:**
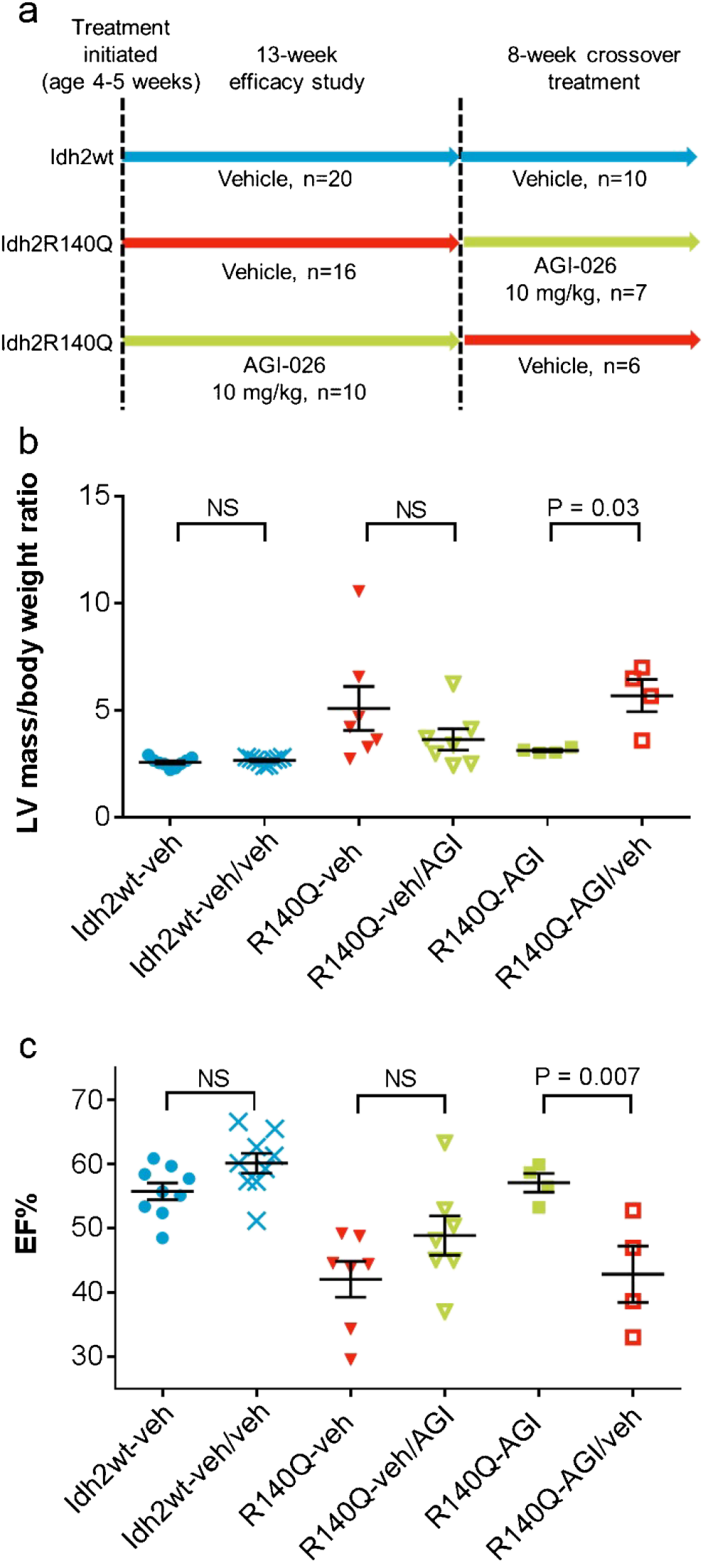
Cross-over study to determine effects of withdrawal of AGI-026 treatment. (**a**) Cross-over study design. (**b**) Left ventricular mass/body weight and (**c**) ejection fraction before and after cross-over study treatment (*n* = 9 in Idh2wt, *n* = 7 in Idh2R140Q-veh/AGI, and *n* = 4 in Idh2R140Q-AGI/veh). Error bars represent mean ± standard deviation. Statistical significance (p) tested with one-way ANOVA with Sidak’s multiple comparison test

To determine if continuous administration of AGI-026 was required to sustain benefit, and if Idh2R140Q inhibition could reverse existing cardiac dysfunction, we implemented treatment cross-over immediately after the second 13-week efficacy study. Idh2R140Q-AGI mice were switched to vehicle (Idh2R140Q-AGI/veh) and Idh2R140Q-veh mice switched to AGI-026 10 mg/kg (Idh2R140Q-veh/AGI) (Fig. 4a). Interim echocardiography 1 month after cross-over showed a trend toward improved cardiac function in Idh2R140Q-veh/AGI mice, with decreases in LVM/BW and increases in EF (not significant; Fig. 4b, c). All Idh2R140Q-AGI/veh mice surviving at this interim time point showed a statistically significant increase in LVM/BW (*p* = 0.03; Fig. 4b) and a decrease in EF (*p* = 0.007; Fig. 4c). After 8 weeks of cross-over treatment, all seven Idh2R140Q-veh/AGI mice survived versus only two of six Idh2R140Q-AGI/veh mice. Thus, withdrawal of AGI-026 resulted in significant deterioration in cardiac function, indicating that continuous administration is necessary to sustain normalization of cardiac function and provide survival benefit.

### DNA hydroxymethylation was decreased in heart tissue of Idh2R140Q mice

**Fig. 5:**
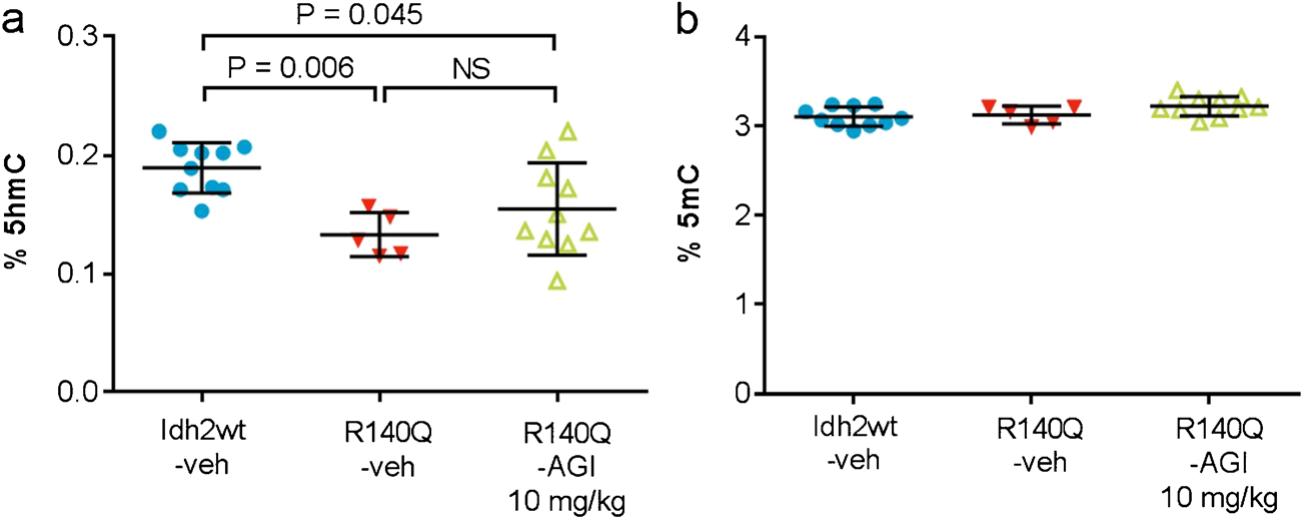
5hmC and 5mC levels in heart tissue of mice. Percentage of 5hmc (**a**) or 5mc (**b**) in hearts of Idh2wt mice receiving vehicle (*n* = 10) and Idh2R140Q mice treated for 13 weeks with vehicle (*n* = 5) or AGI-026 10 mg/kg (*n* = 10). Error bars represent mean ± standard deviation. Statistical significance (p) was tested by one-way ANOVA with Sidak’s multiple comparison test

2HG exerts epigenetic effects by competitive inhibition of αKG-dependent dioxygenases, including histone demethylases (Xu et al 2011; Lu et al 2012), and the ten-eleven-translocation (Tet) enzymes (Xu et al 2011), which mediate conversion of 5-methylcytosine (5mC) into 5-hydroxymethylcytosine (5hmC) (Ito et al 2011). IDH-mutated tumors display DNA hypermethylation, with significantly lower 5hmC and higher 5mC levels versus IDHwt tumors and normal tissues (Figueroa et al 2010; Liu et al 2012; Turcan et al 2012; Wang et al 2013b; Kroeze et al 2014). As there is currently no knowledge of DNA or histone methylation characteristics in D2HGA type II primary human samples, we assessed methylation patterns in heart tissue from mutant versus wt mice. No differences were observed in histone methylation at H3K9 and H3K27. However, %5hmC was significantly lower in Idh2R140Q-veh than in Idh2wt-veh mice (*p* = 0.006), and there was a non-significant trend toward higher %5hmc in Idh2R140Q-AGI versus Idh2R140Q-veh mice (Fig. 5a). Total 5mC was consistent in all three groups (Fig. 5b).

### Gene expression and metabolism are broadly dysregulated in Idh2R140Q heart tissue

To test our hypothesis that the cardiomyopathy observed in Idh2R140Q mice may be associated with systemic metabolic dysregulation, we characterized genetic and metabolic alterations in heart tissue using RNA sequencing and quantitative metabolomics.

**Fig. 6:**
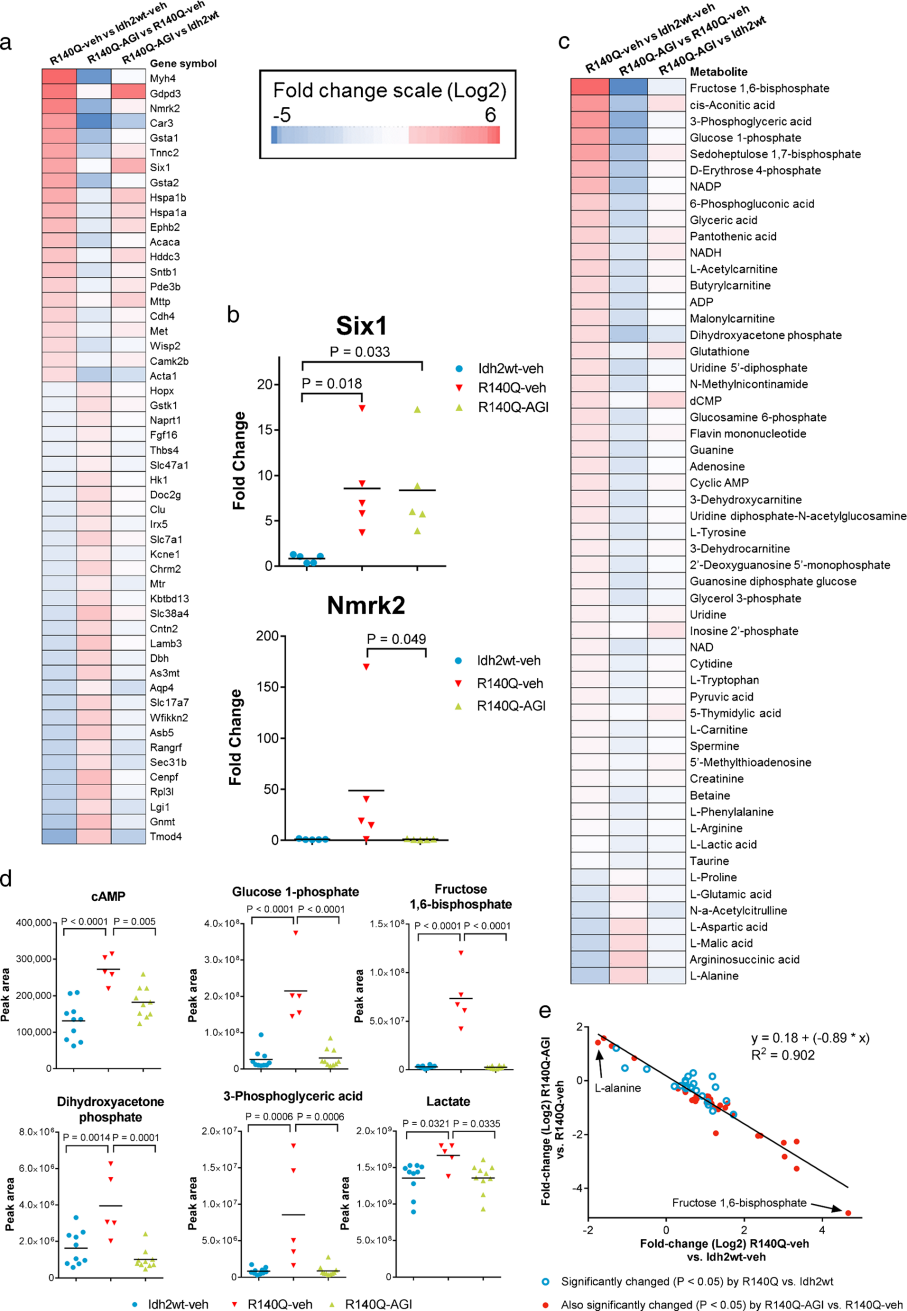
Differences in metabolite levels and gene expression in Idh2R140Q and Idh2wt heart tissue and effect of AGI-026. For heat maps (**a**) and (**c**), red indicates an increase, blue a decrease, darker colors indicate greater change, and pale colors indicate less change. Left column shows difference for Idh2R140Q versus Idh2wt, central column shows difference for AGI-026 versus vehicle treatment in Idh2R140Q mice, and right column shows difference for AGI-026-treated Idh2R140Q versus Idh2wt mice (pale colors illustrate correction of alterations by AGI-026 and shift to Idh2wt status). (**a**) Expression changes in 52 candidate genes with ≥ 2-fold difference between Idh2R140Q (*n* = 6) and Idh2wt (*n* = 5); (Idh2R140Q-AGI *n* = 6). (**b**) Quantitative RT-PCR validation of expression changes in two candidate genes, shown as relative expression normalized to one Idh2wt sample. Each data point represents the mean of a technical triplicate from each of the five animals per group. P tested using Kruskal-Wallis tests with Dunn’s multiple comparison test. For all figures horizontal black bars indicate group means. (**c**) The metabolites (excluding 2HG) that were significantly changed (*p* < 0.05) in heart tissue of Idh2R140Q versus Idh2wt mice, and effects of AGI-026. (**d**) Levels of cAMP and metabolites in the glycogenolysis and glycolysis pathways that are corrected by AGI-026 treatment. Statistical significance (p) was tested with one-way ANOVA with Sidak’s multiple comparison test (*n* = 10 Idh2wt-veh and Idh2R140Q-AGI, *n* = 5 Idh2R140Q-veh). (**e**) Correlation of fold-changes for Idh2R140Q-AGI versus Idh2R140Q-veh against Idh2R140Q-veh versus Idh2wt-veh for the metabolites in heart tissue significantly altered by Idh2R140Q expression (excluding 2HG). *Red dots* indicate metabolites significantly changed on both axes (*n* = 32). R^2^ denotes coefficient of determination

The genes and metabolites showing the greatest changes represent a broad spectrum of metabolic pathways (Supplementary Table 4), indicating widespread metabolic perturbation in the Idh2R140Q mouse heart. Significant differences (*p* < 0.05) in the expression of 174 genes were observed in Idh2R140Q versus Idh2wt heart tissue. Functional annotation revealed significant enrichment in amino acid metabolism and cofactors of cellular functions such as differentiation, cell signaling, antigen processing, and muscle morphology and development (Supplementary Table 5). Three genes contributing to the antigen processing enrichment (GO category 0019886) were upregulated by ∼2-fold in Idh2R140Q mouse hearts (histocompatibility receptors, Entrezgene IDs: 14960, 14961 and 14969). Using natural language processing-based text mining (Friedman et al 2001; Novichkova et al 2003; Jensen et al 2006) in MEDLINE, we sought published causal associations between the differentially expressed genes and cardiomyopathy, cardiomyocyte development and differentiation or dysregulated metabolism, and identified 52 candidates showing at least a twofold change (21 upregulated, 31 downregulated; *p* < 0.05; Fig. 6a). Processes regulated by these genes include cardiac muscle growth and differentiation, muscle structure, regulation of contraction, and regulation of muscle cell metabolism (Supplementary Table 4). Several have been shown to be adversely associated with heart failure, namely the transcription factors HOP homeobox (*Hopx)* (Trivedi et al 2011) and Six homeobox 1 (*Six1*) (Delgado-Olguin et al 2012; Wu et al 2014), the cytoskeletal regulator tropomodulin 4 (*Tmod4*) (Zhao et al 2013), and the metabolic gene nicotinamide riboside kinase 2 (*Nmrk2*) (Ruggieri et al 2015). In Idh2R140Q mouse hearts we observed a twofold downregulation in *Hopx* expression, a 25-fold downregulation in *Tmod4*, a 25-fold upregulation in *Nmrk2*, and an 11-fold upregulation in *Six1*, as well as upregulation of *Six1* downstream targets (Fig. 6a, b; Supplementary Table 6). AGI-026 treatment reversed the majority of these Idh2R140Q-induced changes, with expression of 18 of the 21 upregulated and 23 of the 31 downregulated genes being normalized to levels seen in Idh2wt mice, including *Hopx, Tmod4* and *Nmrk2* (*p* = 0.049) (Fig. 6a, b). AGI-026 treatment failed to reduce *Six1* expression (Fig. 6a, b).

Significant Idh2R140Q-induced differences in levels were observed for 56 of 126 metabolites assessed in heart tissue (*p* < 0.05, Supplementary Table 7). The magnitude of change ranged from 1.2- to 25-fold, with 48 increasing (excluding 2HG) and seven decreasing (Fig. 6c). Notably, in Idh2R140Q-veh compared to Idh2wt-veh hearts we observed decreases in tricarboxylic acid cycle (TCA) intermediates glutamate (43 % reduction; *p* = 0.01), malate (62 % reduction; *p* = 0.03), and aspartate (59 % reduction; *p* = 0.01), as well as elevations in intermediates of glycogenolysis and glycolysis (Fig. 6d), and a twofold increase in the second messenger, cAMP, all of which were normalized with AGI-026 treatment (Fig. 6c, d). Consistent with increased cAMP, we observed a significant increase in expression of its downstream target, phosphodiesterase 3B (Maurice et al 2003) (Pde3b; Fig. 6a). AGI-026 treatment reversed dysregulation in the majority of these significantly altered metabolites in the Idh2R140Q mouse heart (Fig. 6c, d; Supplementary Table 7). Plotting the fold-change for Idh2R140Q-AGI/Idh2R140Q-veh treated mice against fold-change for Idh2R140Q-veh/Idh2wt-veh treated mice yielded a slope of −0.89 and a coefficient of determination of 0.90 (Fig. 6e).

Taken together, these data indicate differential expression of multiple genes and metabolites associated with cardiomyopathy in Idh2R140Q mice, which were largely reversed by AGI-026.

## Discussion

We have generated a novel mouse model of D2HGA type II, a rare neurometabolic disorder caused by germline gain-of-function mutations in *IDH2*. Our *Idh2R140Q* KI model displays dramatically elevated 2HG levels in plasma, bone marrow, heart, spleen, and brain compared with Idh2wt littermates, which is associated with early onset cardiomyopathy, brain abnormalities, shortened life span, and in some mice, periodic seizures, runting, and face/head abnormalities. We demonstrated that treatment with AGI-026, a selective, brain-penetrant IDH2R140Q inhibitor, not only prevented progression of cardiomyopathy in young Idh2R140Q mice but also ameliorated cardiomyopathy in adult animals, and provided a survival benefit in this model, with continuous administration being required to sustain these benefits. We also observed differential expression of multiple genes and metabolites in Idh2R140Q hearts, some of which have been associated with cardiomyopathy, that were largely reversed by AGI-026 treatment. Our findings provide proof-of-concept for the clinical potential of mutant IDH2 inhibition in treating this neurometabolic disorder.

The association of a more severe D2HGA phenotype with the higher 2HG levels observed in type II versus type I disease points to a key role for 2HG in disease pathogenesis. Cardiomyopathy occurs in ∼50 % type II cases but not in type I, and our data show that a 75 % reduction in plasma 2HG is sufficient to ameliorate cardiomyopathy in this mouse model, suggesting that only very high 2HG levels induce this pathology. Notably, wild-type Idh2 mRNA and protein expression is highest in heart tissue. However, cardiomyopathy in D2HGA type II may also be related to NADPH deficiency resulting from consumption of this co-factor in the reduction of αKG to 2HG by mutant IDH2. Alterations in NADP+/NADPH ratios affect intermediary metabolism that may have subsequent effects on cardiac function (Ussher et al 2012). In the heart, NADPH plays a role in fatty acid synthesis and also regulation of oxidative stress through the thioredoxin system, which utilizes NADPH to regulate the redox status of various intracellular proteins, resulting in a cardioprotective effect (Ussher et al 2012).

Given that 2HG inhibits histone and DNA demethylases (Figueroa et al 2010; Chowdhury et al 2011; Xu et al 2011), changes in 5hmC/5mC are expected. We observed significantly lower %5hmC in heart tissue of Idh2R140Q mice, which was increased by AGI-026 treatment, although this did not reach significance in the mouse model, possibly as a result of a less severe phenotype in surviving Idh2R140Q mice. No difference in 5mc levels was observed. Others have shown changes in 5hmC without concomitant 5mC changes in IDH1/2-mutant tissues or cells (Kraus et al 2012; Chen et al 2013), and in spleens of mice engrafted with *IDH2R140Q*-transduced progenitor cells (Mylonas et al 2014). As the 5mC pool is much larger than the 5hmC pool, it is possible that the technique used was not sensitive enough to detect relatively subtle changes in 5mC. Our data suggest that aberrant DNA hydroxymethylation may play a role in cardiomyopathy in this model, consistent with other reports indicating epigenetic changes in human heart failure (Movassagh et al 2011) and the role of epigenetics in cardiac development and homeostasis (Delgado-Olguin et al 2012). Accordingly, we identified differential expression in several genes in Idh2R140Q mouse hearts that, when dysregulated, could be implicated in cardiomyopathy, including downregulation of *Hopx* (Trivedi et al 2011) and *Tmod4* (Zhao et al 2013) and upregulation of *Six1* (Delgado-Olguin et al 2012; Wu et al 2014) and *Nmrk2* (Li et al 1999; Ruggieri et al 2015). Assessment of AGI-026-mediated transcriptional changes in fibroblasts from a patient with D2HGA type II also revealed gene pathways with known relevance in cardiovascular system development and function (including cardiac hypertrophy), and myogenesis (personal communication, MDD and Stéphane de Botton). Cardiomyopathy has been linked to defects in myocardial energy production (Ingwall 2009), increased oxidative stress (Christiansen et al 2015), and structural defects in cardiomyocytes (Harvey and Leinwand 2011). Fatty acids are the primary source of energy in the healthy heart (Strauss and Johnson 1996), but in heart failure there is a shift toward glycolysis (Allard et al 1994). Metabolite profiling of Idh2R140Q mouse heart tissue reflected these observations, with significant increases in levels of glycolytic intermediates and decreases in steady-state levels of the TCA and anaplerotic metabolites, glutamate, malate and aspartate, consistent with observations in human cancer cells expressing IDH1/2 mutations (Reitman et al 2011). Given that these alterations are observed across different tissues, they likely represent a shared metabolic burden of IDH mutation, although it is not yet clear whether they are the cause or consequence of cardiomyopathy. We also observed increases in cAMP; chronic activation of the cAMP/protein kinase A pathway stimulates glycogen breakdown and glycolysis in the heart and is believed to contribute to the pathophysiology of cardiomyopathy (Depre et al 1998; Movsesian 2004). Together, our data show that the Idh2R140Q mutation induces significant epigenetic, transcriptional, and metabolic dysregulation in heart tissue. Our metabolic profiling may be limited by the speed at which we were able to freeze the tissues. Intervals of <5 seconds between life and freezing are the ideal for capturing metabolite status due to significant changes occurring rapidly upon induction of anoxia (Faupel et al 1972; Hearse 1983). Although there is a risk our metabolic profiling was not exhaustive, the results are nevertheless informative as they reflect significant differences between Idh2R140Q mutant, AGI-026 treated, and matching control Idh2wt mice from the same experimental setting, and they are also supported by the expression profiling data.

Patients with D2HGA type II have a limited life span and exhibit a wide spectrum of symptoms including developmental delay, epilepsy, and cardiomyopathy in ∼50 % of cases. Our Idh2R140Q mouse model recapitulates these defects and thus provides a valuable preclinical system to further characterize D2HGA type II and to evaluate approaches for therapeutic intervention. A previously reported transgenic mouse model over-expressing Idh2R140Q or Idh2R172K mutations under post-natal, conditional activation displayed elevated serum 2HG, cardiomyopathy, brain vacuoles and muscular dystrophy, with a more severe phenotype when Idh2R140Q was embryonically activated (including runting, tremors, seizures, hydrocephalus, and shortened lifespan). Improvements in cardiac function and survival were observed on elimination of transgene expression (Akbay et al 2014). This study also noted the same upregulation of gene expression in the antigen processing and presentation category (GO:0019886) that we observed, potentially indicating a connection between Idh2R140Q expression and specific immune responses. The renal abnormalities observed in our model were not described in the transgenic mouse model (Akbay et al 2014), and were not rescued by AGI-026, suggesting that they may be mouse-strain specific. Our metabolic findings were generally consistent with the Idh2R140Q transgenic mouse model (Akbay et al 2014), although there appear to be some differences regarding glycogen and glucose metabolism. However, in the transgenic model, high levels of mutant Idh2 from a non-native promoter were expressed upon tamoxifen administration from 5 weeks of age, whereas our approach introduced the Idh2R140Q mutation at the endogenous chromosomal locus, resulting in physiological regulation of expression beginning in embryogenesis. Consequently, our model reflects the genetic, developmental, and physiological circumstances of the disease in humans. The suitability of our model for early administration of experimental small molecule inhibitors also carries implications for future clinical intervention and prevention.

The observations that the IDH2R140Q inhibitor, AGI-026, rescued cardiomyopathy and provided a survival benefit in this model through 2HG reduction, and potently inhibited 2HG production in lymphoblasts from D2HGA patients carrying IDH2R140Q mutations, suggest that mutant IDH2 inhibition has therapeutic potential in D2HGA type II. Importantly, administration of AGI-026 largely reversed the transcriptional and metabolic alterations in heart tissue, providing insight into the pathophysiology of cardiomyopathy in D2HGA type II. Further investigation is warranted to determine the molecular mechanisms behind these changes.

## Electronic supplementary material

Below is the link to the electronic supplementary material.

ESM 1 (DOCX 309 kb)
