# Supplementary material for: A small molecule inhibitor of mutant IDH2 rescues cardiomyopathy in a D-2-hydroxyglutaric aciduria type II mouse model
Source: J Inherit Metab Dis. 2016 Jul 28;39(6):807–20. doi: 10.1007/s10545-016-9960-y (PMC5065612; doi:10.1007/s10545-016-9960-y)

**­­­­­­Supplementary material**

**A small molecule inhibitor of mutant IDH2 rescues cardiomyopathy in a D-2-hydroxyglutaric aciduria type II mouse model**

Fang Wang, Jeremy Travins, Zhizhong Lin, Yaguang Si, Yue Chen, Josh Powe, Stuart Murray, Dongwei Zhu, Erin Artin, Stefan Gross, Stephanie Santiago, Mya Steadman, Andrew Kernytsky, Kimberly Straley, Chenming Lu, Ana Pop, Eduard A Struys, Erwin Jansen, Gajja S Salomons, Muriel D David, Cyril Quivoron, Virginie Penard-Lacronique, Karen S Regan, Wei Liu, Lenny Dang, Hua Yang, Lee Silverman, Samuel Agresta, Marion Dorsch, Scott Biller, Katharine Yen, Yong Cang, Shin-San Michael Su and Shengfang Jin

**Neurological testing in Idh2R140Q mice**

The pathological significance of the vacuoles observed in numerous brain structures of Idh2R140Q mice is not yet clear, but based on the brain regions involved, we suspected that learning, memory, recognition and motor functions, among others, could be disrupted. We therefore performed preliminary neurological tests including grip strength, rotarod performance, local motor activity, and novel object recognition (NOR), and observed hind limb weakness and significantly shorter fall-off time at 16 rpm in Idh2R140Q mice, but no difference in NOR. A more detailed analysis of developmental delay is warranted using this model.

**Supplementary Table 1**

Heart and kidney variables in Idh2wt and Idh2R140Q KI mice. Values shown are mean (SD)

| **Parameter** | **Idh2wt** | **Idh2R140Q** |
| --- | --- | --- |
| Heart weight g, mean (SD) | 0.155 (0.02) | 0.214 (0.03) |
| Heart weight/body weight ratio, mean (SD) | 5.76 (0.24) | 8.08 (1.14) |
| Left ventricular mass/body weight ratio, mean (SD) | 2.67 (0.22) | 4.58 (1.89) |
| Ejection fraction %, mean (SD) | 56.39 (3.16) | 44.03 (7.87) |
| Blood urea nitrogen nmol/L, mean | 14.73 | 20.73 |
| Creatinine μmol/L, mean | 6 | 17 |
| Kidney weight g, mean (SD) | 0.3785 (0.03) | 0.5989 (0.24) |
| Kidney/body weight ratio, mean (SD) | 0.0141 (0.0003) | 0.0226 (0.01) |
| Kidney/brain weight ratio, mean (SD) | 0.7955 (0.06) | 1.3066 (0.58) |

**Supplementary Table 2**

Heart histopathology findings from individual Idh2R140Q mice treated with vehicle or AGI-026 10 mg/kg. F/M denotes female/male

| **Group** | **Animal** | **Minimal myocardial hypertrophy** | **Minimal cardiomegaly** |
| --- | --- | --- | --- |
| Idh2R140Q-veh | 31F |  |  |
|  | 33F | x |  |
|  | 34F | x | x |
|  | 45M | x | x |
|  | 46M | x | x |
|  | 47M | x | x |
| Total: | | 5 | 4 |
| Idh2R140Q-AGI  10 mg/kg | 51F |  |  |
|  | 54F | x |  |
|  | 55F |  |  |
|  | 63M |  |  |
|  | 65M |  |  |
|  | 66M |  |  |
| Total: | | 1 | 0 |

**Supplementary Table 3**

Brain histopathology findings from individual Idh2R140Q mice treated with vehicle or AGI-026 10 mg/kg – vacuolated areas

| **Group** | **Animal** | **Lateral septal nuclei** | **Motor cortex** | **Somato-sensory cortex** | **Piriform cortex** | **Retrosplenial cortex** | **Anterior cingulate cortex** | **Hippocampus** | **Dentate gyrus** | **Amygdala** | **Medulla** | **Total** |
| --- | --- | --- | --- | --- | --- | --- | --- | --- | --- | --- | --- | --- |
| Idh2wt-veh | 1F |  |  |  |  |  |  |  |  |  |  | 0 |
|  | 5F |  |  |  |  |  |  |  |  |  |  | 0 |
|  | 6F |  |  |  |  |  |  |  |  |  |  | 0 |
|  | 21M |  |  |  |  |  |  |  |  |  |  | 0 |
|  | 24M |  |  |  |  |  |  |  |  |  |  | 0 |
|  | 25M | x | x | x |  | x | x | x | x |  |  | 7 |
| Average number of sites affected | | | | | | | | | | | | 1.2 |
| Idh2R140Q  -veh | 31F | x | x | x |  | x | x | x | x |  | x | 8 |
|  | 33F |  |  |  |  |  |  | x |  |  |  | 1 |
|  | 34F | x |  |  |  |  |  | x | x | x |  | 4 |
|  | 45M | x | x | x |  | x |  | x | x | x | x | 8 |
|  | 46M | x | x | x |  | x | x | x | x |  |  | 7 |
|  | 47M |  |  |  |  |  |  |  |  |  |  | 0 |
| Average number of sites affected | | | | | | | | | | | | 4.7 |
| Idh2R140Q  -AGI  10 mg/kg | 51F |  | x |  |  |  |  | x |  |  |  | 2 |
|  | 54F | x |  |  |  |  |  |  | x |  |  | 2 |
|  | 55F |  |  |  |  |  |  |  |  |  |  | 0 |
|  | 63M |  | x |  | x |  |  | x |  |  |  | 3 |
|  | 65M |  |  |  |  | x |  | x |  |  | x | 3 |
|  | 66M |  |  |  |  | x | x |  | x |  |  | 3 |
| Average number of sites affected | | | | | | | | | | | | 2.2 |

**Supplementary Table 4**

Biological pathways showing the greatest changes based on differential gene expression/metabolite levels

| **Process** | **Differential genes/*metabolites*** |
| --- | --- |
| **Cardiac/muscle processes** |  |
| Cardiac muscle growth and differentiation | Asb5, Fgf16, Tmod4, Wfikkn2 |
| Muscle structure and regulation of contraction | Acta1, Myh4, Sntb1, Tnnc2, Chrm2, Kcne1 |
| Muscle cell metabolism | Aqp4, Dbh, Doc2g, Slc47a1, Thbs4 |
| Pathology of heart failure | As3mt, Lamb3 |
| **Metabolism** |  |
| Glycolysis | Hk1, *Fructose 1,6-biphosphate, Dihydroxyacetone phosphate, 3-Phosphoglyceric acid, Glyceric acid* |
| TCA | *cis-Aconic acid, Malate* |
| Pentose phosphate pathway | *6-Phosphogluconic acid and D-Erythrose 4-phosphate* |
| Glycogen metabolism | *Glucose 1-phosphate* |
| Lipid synthesis | Acaca |
| Lipid degradation | Gdpd3, Pde3b, *short-chain carnitines (C0~C4)* |
| Lipid transport | Mttp |
| Methionine and 1-carbon pathway | Gnmt, Mtr |
| NAD synthesis/metabolism | Naprt1, Nmrk2, *NADP, NADH*, |
| Nucleotide metabolism/second messenger | *UDP,* *Adenosine, Guanine, dCMP, cyclic AMP, cAMP* |
| Energy/redox | Gsta1, Gsta2, Gstk1, Car3, Clu, Hspa1a, Hspa1b, *ADP, N-Methylnicotinamide, Reduced glutathione, Flavin Mononucleotide* |
| Vitamin/cofactors | *Pantothenic acid* |
| Amino- and nucleotide-sugars | *Glucosamine 6-phosphate and ADP-glucose* |
| **Cell physiology** |  |
| Organ growth and differentiation | Camk2b, Cenpf, Cntn2, Ephb2 |
| Cell adhesion and communication | Cdh4, Lgi1, Slc17a7, Wisp2 |
| Mitochondrial homeostasis | Slc38a4 |
| Synthesis, degradation and trafficking of proteins | Kbtbd13, Rangrf, Rpl3l Sec31b |

**Supplementary Table 5**

Pathway enrichment of differentially expressed genes in Idh2R140Q compared with Idh2wt hearts

| **Category** | **Term** | **Fold enrichment** | **P value** |
| --- | --- | --- | --- |
| GO:0006928 | Cell motion | 3.20 | 3.90E-03 |
| GO:0051186 | Cofactor metabolic process | 4.52 | 4.44E-03 |
| GO:0051239 | Regulation of multicellular organismal process | 2.27 | 5.88E-03 |
| GO:0006575 | Cellular amino acid derivative metabolic process | 5.00 | 6.85E-03 |
| GO:0006732 | Coenzyme metabolic process | 4.93 | 7.26E-03 |
| GO:0002495 | Antigen processing and presentation of peptide antigen via MHC class II | 22.03 | 7.85E-03 |
| GO:0019886 | Antigen processing and presentation of exogenous peptide antigen via MHC class II | 22.03 | 7.85E-03 |
| GO:0006790 | Sulfur metabolic process | 6.25 | 8.21E-03 |
| GO:0050793 | Regulation of developmental process | 2.48 | 8.55E-03 |
| GO:0048699 | Generation of neurons | 2.60 | 9.35E-03 |
| GO:0002504 | Antigen processing and presentation of peptide or polysaccharide antigen via MHC class II | 18.55 | 1.10E-02 |
| GO:0051179 | Localization | 1.50 | 1.15E-02 |
| GO:0022008 | Neurogenesis | 2.41 | 1.54E-02 |
| GO:0002478 | Antigen processing and presentation of exogenous peptide antigen | 15.33 | 1.59E-02 |
| GO:0016477 | Cell migration | 3.43 | 1.61E-02 |
| GO:0045597 | Positive regulation of cell differentiation | 4.03 | 1.63E-02 |
| GO:0007517 | Muscle organ development | 4.01 | 1.67E-02 |
| GO:0006749 | Glutathione metabolic process | 14.69 | 1.73E-02 |
| GO:0030154 | Cell differentiation | 1.65 | 2.17E-02 |
| GO:0048523 | Negative regulation of cellular process | 1.77 | 2.23E-02 |
| GO:0007519 | Skeletal muscle tissue development | 6.53 | 2.29E-02 |
| GO:0019884 | Antigen processing and presentation of exogenous antigen | 12.59 | 2.31E-02 |
| GO:0060538 | Skeletal muscle organ development | 6.35 | 2.46E-02 |
| GO:0044057 | Regulation of system process | 3.51 | 2.77E-02 |
| GO:0048869 | Cellular developmental process | 1.58 | 3.25E-02 |
| GO:0006066 | Alcohol metabolic process | 2.61 | 3.29E-02 |
| GO:0051674 | Localization of cell | 2.90 | 3.33E-02 |
| GO:0048870 | Cell motility | 2.90 | 3.33E-02 |
| GO:0040011 | Locomotion | 2.58 | 3.46E-02 |
| GO:0051094 | Positive regulation of developmental process | 3.29 | 3.50E-02 |
| GO:0048002 | Antigen processing and presentation of peptide antigen | 10.07 | 3.51E-02 |
| GO:0001655 | Urogenital system development | 4.02 | 3.51E-02 |
| GO:0006519 | Cellular amino acid and derivative metabolic process | 2.77 | 4.00E-02 |
| GO:0006518 | Peptide metabolic process | 9.28 | 4.08E-02 |
| GO:0051188 | Cofactor biosynthetic process | 5.17 | 4.17E-02 |
| GO:0044271 | Nitrogen compound biosynthetic process | 2.72 | 4.28E-02 |
| GO:0045595 | Regulation of cell differentiation | 2.44 | 4.51E-02 |
| GO:0007275 | Multicellular organismal development | 1.40 | 4.54E-02 |
| GO:0060284 | Regulation of cell development | 3.70 | 4.57E-02 |
| GO:0060638 | Mesenchymal-epithelial cell signaling | 39.17 | 4.96E-02 |
| GO:0048013 | Ephrin receptor signaling pathway | 39.17 | 4.96E-02 |
| GO:0014812 | Muscle cell migration | 39.17 | 4.96E-02 |

**Supplementary Table 6**

Changes in expression of *Six1* and downstream gene targets in Idh2R140Q (n=6, 3 male, 3 female) versus Idh2wt (n=5, 3 male, 2 female) mice

| **Name** | **Description** | **Log2FC Idh2R140Q versus Idh2wt** | **P value** |
| --- | --- | --- | --- |
| MYH4 | Myosin, heavy chain 4, skeletal muscle | 4.435 | 4.0E-02 |
| SIX1 | SIX homeobox 1 | 3.3447 | 6.9E-04 |
| ATP2A1 | ATPase, Ca++ transporting, cardiac muscle, fast twitch 1 | 2.4741 | 2.9E-02 |
| MYLPF | Myosin light chain, phosphorylatable, fast skeletal muscle | 2.2649 | 3.5E-02 |
| PVALB | Parvalbumin | 1.6003 | 7.5E-02 |
| NEUROD1 | Neurogenic differentiation 1 | 1.5832 | 2.5E-02 |
| MYC | v-myc myelocytomatosis viral oncogene homolog (avian) | 1.383 | 8.7E-02 |
| OTX2 | Orthodenticle homeobox 2 | 1.0863 | 5.2E-02 |
| MYOZ1 | Myozenin 1 | 1.0743 | 2.0E-01 |
| PAX2 | Paired box 2 | 1.0562 | 1.6E-01 |
| MYF5 | Myogenic factor 5 | 0.9372 | 3.4E-02 |
| FGF10 | Fibroblast growth factor 10 | 0.8799 | 2.7E-01 |
| ATOH1 | Atonal homolog 1 (Drosophila) | 0.8146 | 2.7E-03 |
| NEUROG1 | Neurogenin 1 | 0.6569 | 1.5E-01 |
| PAX8 | Paired box 8 | 0.6173 | 5.4E-02 |
| SHH | Sonic hedgehog | 0.5911 | 3.0E-01 |
| SIX4 | SIX homeobox 4 | 0.401 | 3.3E-02 |
| CCNA2 | Cyclin A2 | 0.3869 | 3.9E-04 |
| E2F1 | E2F transcription factor 1 | 0.3768 | 4.0E-02 |
| LFNG | LFNG O-fucosylpeptide 3-beta-N-acetylglucosaminyltransferase | 0.3409 | 2.8E-01 |
| MYF6 | Myogenic factor 6 (herculin) | 0.3048 | 8.1E-01 |
| WNT4 | Wingless-type MMTV integration site family, member 4 | 0.2815 | 7.1E-01 |
| IGFBP5 | Insulin-like growth factor binding protein 5 | 0.247 | 5.3E-01 |
| EYA2 | Eyes absent homolog 2 (Drosophila) | 0.2456 | 1.6E-01 |
| PAX3 | Paired box 3 | 0.2084 | 7.2E-01 |
| HOXA3 | Homeobox A3 | 0.1155 | 8.6E-01 |
| ALDOA | Aldolase A, fructose-bisphosphate | 0.0999 | 1.1E-01 |
| SMAD3 | SMAD family member 3 | 0.0645 | 3.7E-01 |
| SLC12A2 | Solute carrier family 12 (sodium/potassium/chloride transporters), member 2 | 0.0328 | 8.0E-01 |
| NOTCH1 | Notch 1 | -0.0188 | 7.1E-01 |
| CCND1 | Cyclin D1 | -0.0242 | 9.2E-01 |
| BMP4 | Bone morphogenetic protein 4 | -0.1109 | 7.2E-01 |
| SALL1 | Sal-like 1 (Drosophila) | -0.1214 | 3.6E-01 |
| MYOG | Myogenin (myogenic factor 4) | -0.145 | 8.2E-01 |
| FOXI1 | Forkhead box I1 | -0.156 | 7.8E-01 |
| CCNA1 | Cyclin A1 | -0.1884 | 8.5E-01 |
| VEGFC | Vascular endothelial growth factor C | -0.1974 | 2.1E-04 |
| PAX6 | Paired box 6 | -0.2014 | 4.2E-01 |
| EZR | Ezrin | -0.2851 | 2.9E-01 |
| SOX2 | SRY (sex determining region Y)-box 2 | -0.5666 | 2.4E-01 |
| SIX2 | SIX homeobox 2 | -0.5897 | 6.7E-02 |
| GDNF | Glial cell derived neurotrophic factor | -0.5902 | 1.5E-01 |
| TCAP | Titin-cap (telethonin) | -0.6382 | 7.2E-03 |
| GBX2 | Gastrulation brain homeobox 2 | -0.6877 | 2.3E-01 |
| MYOD1 | Myogenic differentiation 1 | -0.7484 | 3.4E-02 |
| GATA3 | GATA binding protein 3 | -0.8171 | 1.1E-02 |
| OTX1 | Orthodenticle homeobox 1 | -0.9254 | 8.5E-02 |
| EYA1 | Eyes absent homolog 1 (Drosophila) | -0.9574 | 3.8E-04 |
| NEUROG2 | Neurogenin 2 | -1.2465 | 1.2E-01 |
| PHOX2A | Paired-like homeobox 2a | -1.2973 | 1.0E-02 |
| CASQ1 | Calsequestrin 1 (fast-twitch, skeletal muscle) | -1.6711 | 1.1E-04 |
| HMX3 | H6 family homeobox 3 | -1.8114 | 3.1E-01 |

**Supplementary Table 7**

Relative metabolite levels in Idh2wt (n=10) versus Idh2R140Q (n=5) hearts and Idh2R140Q hearts treated with AGI-026 (n=10)

| **HMDB** | **Metabolite** | **Fold: R140Q-veh versus wt-veh** | **p Value: R140Q-veh versus wt-veh** | **Fold: R140Q-AGI versus R140Q-veh** | **p Value: R140Q-AGI versus R140Q-veh** |
| --- | --- | --- | --- | --- | --- |
| HMDB00001 | 1-Methylhistidine | 1.01 | 9.8E-01 | 1.08 | 7.3E-01 |
| HMDB01294 | 2,3-Diphosphoglyceric acid | 10.46 | 9.0E-02 | 0.51 | 4.8E-01 |
| HMDB01044 | 2'-Deoxyguanosine 5'-monophosphate | 1.70 | 3.7E-03 | 0.67 | 2.9E-02 |
| HMDB12154 | 3-Dehydrocarnitine | 1.71 | 3.6E-03 | 0.77 | 1.9E-01 |
| HMDB06831 | 3-Dehydroxycarnitine | 2.04 | 9.9E-04 | 0.57 | 3.4E-03 |
| HMDB61636 | 3-hydroxydecanoyl carnitine | 0.71 | 3.0E-01 | 1.54 | 2.8E-01 |
| HMDB01861 | 3-Methylhistamine | 0.72 | 2.7E-01 | 2.64 | 5.9E-02 |
| HMDB00807 | 3-Phosphoglyceric acid | 10.14 | 4.1E-03 | 0.10 | 4.5E-03 |
| HMDB06055 | 4-Hydroxy-L-proline | 0.79 | 2.2E-01 | 1.41 | 1.2E-01 |
| HMDB01173 | 5'-Methylthioadenosine | 1.41 | 2.8E-03 | 1.01 | 9.5E-01 |
| HMDB01227 | 5-Thymidylic acid | 1.48 | 4.1E-02 | 1.12 | 8.6E-01 |
| HMDB01316 | 6-Phosphogluconic acid | 3.31 | 1.4E-02 | 0.42 | 5.3E-02 |
| HMDB00034 | Adenine | 1.47 | 6.2E-02 | 0.70 | 9.4E-02 |
| HMDB00050 | Adenosine | 2.08 | 1.1E-02 | 0.58 | 7.9E-02 |
| HMDB00045 | Adenosine monophosphate | 1.21 | 1.0E-01 | 0.80 | 1.8E-01 |
| HMDB01341 | ADP | 2.52 | 5.0E-03 | 0.48 | 4.9E-02 |
| HMDB00462 | Allantoin | 0.99 | 9.7E-01 | 1.14 | 6.7E-01 |
| HMDB00510 | Aminoadipic acid | 0.61 | 1.4E-01 | 2.05 | 3.1E-01 |
| HMDB12189 | Aminopropylcadaverine | 1.11 | 1.3E-01 | 0.86 | 7.6E-02 |
| HMDB00052 | Argininosuccinic acid | 0.33 | 9.8E-03 | 2.99 | 1.9E-03 |
| HMDB00043 | Betaine | 1.40 | 1.7E-02 | 0.74 | 4.7E-02 |
| HMDB02013 | Butyrylcarnitine | 2.61 | 2.5E-04 | 0.53 | 7.9E-03 |
| HMDB01564 | CDP-Ethanolamine | 1.20 | 7.4E-02 | 0.99 | 9.4E-01 |
| HMDB00097 | Choline | 1.22 | 2.5E-01 | 1.06 | 8.4E-01 |
| HMDB00072 | cis-Aconitic acid | 10.14 | 1.2E-05 | 0.21 | 3.6E-04 |
| HMDB00094 | Citric acid | 2.59 | 5.3E-02 | 0.63 | 3.8E-01 |
| HMDB00904 | Citrulline | 0.94 | 7.3E-01 | 1.22 | 4.1E-01 |
| HMDB00064 | Creatine | 1.17 | 2.0E-01 | 0.92 | 5.8E-01 |
| HMDB00562 | Creatinine | 1.40 | 1.0E-02 | 0.97 | 7.7E-01 |
| HMDB00058 | Cyclic AMP | 2.07 | 1.3E-04 | 0.67 | 1.5E-03 |
| HMDB00089 | Cytidine | 1.56 | 7.1E-04 | 0.82 | 2.6E-01 |
| HMDB00095 | Cytidine monophosphate | 1.41 | 9.1E-02 | 0.89 | 7.5E-01 |
| HMDB00606 | D-2-Hydroxyglutaric acid | 110.99 | 3.1E-18 | 0.00 | 3.4E-09 |
| HMDB01202 | dCMP | 2.26 | 1.8E-03 | 1.11 | 9.0E-01 |
| HMDB00651 | Decanoylcarnitine | 0.96 | 9.2E-01 | 1.19 | 5.7E-01 |
| HMDB01321 | D-Erythrose 4-phosphate | 5.37 | 2.0E-05 | 0.24 | 4.1E-05 |
| HMDB00122 | D-Glucose | 0.90 | 7.0E-01 | 1.19 | 5.8E-01 |
| HMDB01473 | Dihydroxyacetone phosphate | 2.41 | 5.2E-03 | 0.26 | 3.0E-04 |
| HMDB00092 | Dimethylglycine | 0.91 | 3.6E-01 | 0.99 | 9.3E-01 |
| HMDB01548 | D-Ribose 5-phosphate | 1.29 | 1.6E-01 | 1.33 | 4.5E-01 |
| HMDB01409 | dUMP | 1.21 | 1.6E-01 | 0.81 | 1.8E-01 |
| HMDB01520 | Flavin Mononucleotide | 2.11 | 1.0E-05 | 0.66 | 4.0E-02 |
| HMDB01058 | Fructose 1,6-bisphosphate | 25.28 | 2.2E-06 | 0.03 | 2.1E-06 |
| HMDB01254 | Glucosamine 6-phosphate | 2.24 | 3.8E-05 | 0.56 | 9.8E-04 |
| HMDB01586 | Glucose 1-phosphate | 8.18 | 3.3E-05 | 0.14 | 3.8E-05 |
| HMDB00125 | Glutathione | 2.39 | 9.0E-03 | 0.85 | 6.3E-01 |
| HMDB00139 | Glyceric acid | 3.30 | 2.3E-04 | 0.41 | 1.0E-03 |
| HMDB01270 | Glyceric acid 1,3-biphosphate | 10.46 | 9.0E-02 | 0.51 | 4.8E-01 |
| HMDB00126 | Glycerol 3-phosphate | 1.63 | 2.6E-03 | 0.61 | 2.4E-03 |
| HMDB00123 | Glycine | 1.92 | 2.0E-01 | 0.90 | 8.0E-01 |
| HMDB00128 | Guanidoacetic acid | 0.70 | 1.1E-01 | 1.32 | 2.4E-01 |
| HMDB00132 | Guanine | 2.11 | 2.5E-02 | 0.54 | 1.2E-01 |
| HMDB03351 | Guanosine diphosphate glucose | 1.68 | 3.6E-03 | 0.58 | 2.9E-03 |
| HMDB00705 | Hexanoylcarnitine | 1.88 | 5.4E-02 | 0.69 | 2.5E-01 |
| HMDB00870 | Histamine | 1.41 | 1.3E-01 | 1.12 | 6.0E-01 |
| HMDB00725 | Hydroxyproline | 0.92 | 7.2E-01 | 1.26 | 3.0E-01 |
| HMDB11681 | Inosine 2'-phosphate | 1.61 | 8.2E-03 | 1.23 | 5.2E-01 |
| HMDB00688 | Isovalerylcarnitine | 1.42 | 9.6E-02 | 0.81 | 2.7E-01 |
| HMDB00201 | L-Acetylcarnitine | 2.84 | 1.1E-06 | 0.49 | 2.7E-04 |
| HMDB00161 | L-Alanine | 0.30 | 8.8E-04 | 2.67 | 7.3E-06 |
| HMDB00517 | L-Arginine | 1.29 | 4.8E-02 | 0.88 | 3.7E-01 |
| HMDB00168 | L-Asparagine | 1.27 | 1.2E-01 | 0.89 | 3.2E-01 |
| HMDB00191 | L-Aspartic acid | 0.41 | 8.0E-03 | 2.32 | 1.5E-01 |
| HMDB00062 | L-Carnitine | 1.43 | 7.9E-03 | 0.83 | 1.7E-01 |
| HMDB00099 | L-Cystathionine | 0.55 | 1.4E-01 | 3.77 | 3.9E-01 |
| HMDB03349 | L-Dihydroorotic acid | 0.96 | 9.3E-01 | 1.30 | 5.8E-01 |
| HMDB00148 | L-Glutamic acid | 0.57 | 1.4E-02 | 1.79 | 1.6E-03 |
| HMDB00641 | L-Glutamine | 0.91 | 7.0E-01 | 0.99 | 9.6E-01 |
| HMDB00177 | L-Histidine | 0.82 | 6.4E-01 | 1.40 | 3.2E-01 |
| HMDB00172 | L-Isoleucine | 0.88 | 3.6E-01 | 1.17 | 3.9E-01 |
| HMDB00684 | L-Kynurenine | 0.13 | 1.5E-01 | 6.45 | 3.2E-01 |
| HMDB00190 | L-Lactic acid | 1.23 | 1.7E-02 | 0.81 | 1.1E-02 |
| HMDB00687 | L-Leucine | 0.99 | 9.3E-01 | 1.01 | 9.1E-01 |
| HMDB00182 | L-Lysine | 1.09 | 3.8E-01 | 1.02 | 8.8E-01 |
| HMDB00156 | L-Malic acid | 0.38 | 2.6E-02 | 2.44 | 3.8E-02 |
| HMDB00696 | L-Methionine | 1.17 | 2.2E-01 | 0.99 | 9.4E-01 |
| HMDB00791 | L-Octanoylcarnitine | 1.53 | 3.7E-01 | 0.89 | 7.5E-01 |
| HMDB00159 | L-Phenylalanine | 1.40 | 5.3E-03 | 0.79 | 1.9E-02 |
| HMDB00162 | L-Proline | 0.71 | 3.3E-02 | 1.35 | 3.0E-01 |
| HMDB00187 | L-Serine | 1.03 | 8.6E-01 | 1.11 | 5.7E-01 |
| HMDB00167 | L-Threonine | 0.86 | 1.7E-01 | 1.28 | 1.4E-02 |
| HMDB00929 | L-Tryptophan | 1.55 | 2.8E-02 | 0.83 | 6.1E-01 |
| HMDB00158 | L-Tyrosine | 1.72 | 1.0E-03 | 0.62 | 4.6E-03 |
| HMDB00883 | L-Valine | 0.91 | 4.8E-01 | 1.17 | 3.5E-01 |
| HMDB02095 | Malonylcarnitine | 2.43 | 5.9E-04 | 0.49 | 2.9E-03 |
| HMDB00202 | Methylmalonic acid | 0.81 | 8.8E-02 | 1.13 | 4.1E-01 |
| HMDB01276 | N1-Acetylspermidine | 0.98 | 9.6E-01 | 1.32 | 4.7E-01 |
| HMDB01186 | N1-Acetylspermine | 0.79 | 5.5E-01 | 1.17 | 5.1E-01 |
| HMDB00856 | N-a-Acetylcitrulline | 0.48 | 3.4E-02 | 1.39 | 6.2E-01 |
| HMDB01067 | N-Acetylaspartylglutamic acid | 1.20 | 7.5E-01 | 6.78 | 3.2E-01 |
| HMDB02817 | N-Acetylglucosamine 6-phosphate | 1.25 | 5.8E-01 | 1.31 | 6.8E-01 |
| HMDB01138 | N-Acetylglutamic acid | 1.64 | 4.1E-01 | 0.94 | 9.2E-01 |
| HMDB00812 | N-Acetyl-L-aspartic acid | 1.91 | 3.0E-01 | 1.05 | 9.5E-01 |
| HMDB00230 | N-Acetylneuraminic acid | 1.45 | 2.0E-01 | 1.73 | 4.0E-01 |
| HMDB03357 | N-Acetylornithine | 0.86 | 3.8E-01 | 1.05 | 7.5E-01 |
| HMDB00902 | NAD | 1.58 | 3.9E-06 | 0.60 | 1.4E-02 |
| HMDB01487 | NADH | 2.90 | 3.0E-02 | 0.59 | 1.4E-01 |
| HMDB00217 | NADP | 5.10 | 2.5E-10 | 0.24 | 1.5E-07 |
| HMDB03152 | N-Methylnicotinamide | 2.27 | 1.4E-03 | 0.52 | 3.8E-03 |
| HMDB00214 | Ornithine | 0.77 | 2.2E-01 | 1.37 | 3.1E-01 |
| HMDB03337 | Oxidized glutathione | 0.86 | 1.1E-01 | 1.26 | 2.3E-02 |
| HMDB00208 | Oxoglutaric acid | 0.94 | 8.0E-01 | 2.20 | 1.9E-01 |
| HMDB00210 | Pantothenic acid | 2.97 | 7.4E-06 | 0.55 | 9.2E-03 |
| HMDB00824 | Propionylcarnitine | 0.66 | 1.4E-01 | 1.49 | 2.1E-01 |
| HMDB00017 | Pyridoxic acid | 1.14 | 5.5E-01 | 1.32 | 5.2E-01 |
| HMDB00243 | Pyruvic acid | 1.55 | 1.7E-05 | 0.85 | 1.2E-01 |
| HMDB00939 | S-Adenosylhomocysteine | 1.05 | 8.4E-01 | 1.50 | 2.8E-02 |
| HMDB01185 | S-Adenosylmethionine | 1.27 | 1.1E-01 | 0.88 | 2.8E-01 |
| HMDB60274 | Sedoheptulose 1,7-bisphosphate | 8.03 | 1.1E-06 | 0.20 | 6.1E-05 |
| HMDB01068 | Sedoheptulose 7-phosphate | 0.97 | 9.3E-01 | 1.46 | 4.8E-01 |
| HMDB01257 | Spermidine | 1.28 | 1.1E-01 | 0.91 | 5.7E-01 |
| HMDB01256 | Spermine | 1.41 | 4.2E-02 | 0.82 | 1.1E-01 |
| HMDB00254 | Succinic acid | 1.10 | 7.2E-01 | 0.81 | 4.1E-01 |
| HMDB00251 | Taurine | 1.16 | 3.1E-02 | 0.87 | 9.6E-02 |
| HMDB00896 | Taurodeoxycholic acid | 2.47 | 2.5E-01 | 0.39 | 2.5E-01 |
| HMDB02366 | Tiglylcarnitine | 1.15 | 4.5E-01 | 0.87 | 5.4E-01 |
| HMDB00300 | Uracil | 0.99 | 9.5E-01 | 0.97 | 8.8E-01 |
| HMDB00828 | Ureidosuccinic acid | 1.69 | 1.2E-01 | 0.96 | 9.3E-01 |
| HMDB00289 | Uric acid | 0.77 | 5.3E-01 | 1.18 | 5.8E-01 |
| HMDB00296 | Uridine | 1.62 | 9.6E-04 | 0.92 | 6.2E-01 |
| HMDB00295 | Uridine 5'-diphosphate | 2.28 | 1.8E-02 | 0.46 | 5.0E-02 |
| HMDB00288 | Uridine 5'-monophosphate | 1.48 | 5.8E-02 | 0.76 | 3.0E-01 |
| HMDB00286 | Uridine diphosphate glucose | 1.35 | 6.0E-02 | 0.93 | 7.5E-01 |
| HMDB00935 | Uridine diphosphate glucuronic acid | 7.69 | 1.1E-01 | 0.28 | 2.2E-01 |
| HMDB00290 | Uridine diphosphate-N-acetylglucosamine | 1.89 | 5.0E-03 | 0.67 | 1.4E-01 |
| HMDB00299 | Xanthosine | 1.09 | 6.9E-01 | 0.98 | 9.5E-01 |

**Supplementary Fig. 1** Generation of D-2-hydroxyglutaric aciduria type II mouse model.

(**a**) Generation of mouse model by insertion of the Idh2R140Q mutation into the native exon 4 locus using a lox-stop-lox cassette. (**b**) and (**c**) confirmation of systemic Idh2R140Q/wt genotype by PCR genotyping and sequencing of PCR products


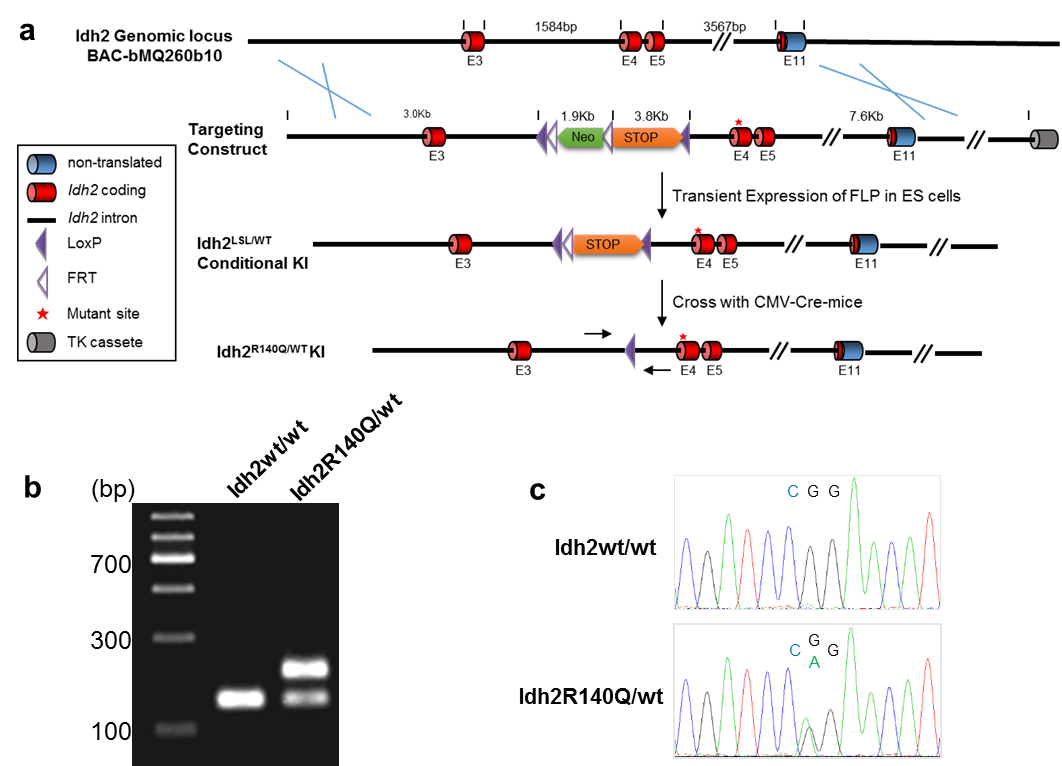


**Supplementary Fig. 2** Characterization of the mouse model.

(**a**) Mendelian ratio for Idh2R140Q KI mice. (**b**) Body weight by sex and time since birth in Idh2wt and Idh2R140Q mice. Error bars indicate the mean ± standard deviation. Statistical significance (p) was tested with one-way ANOVA with Sidak’s multiple comparison test (n=7 Idh2wt male, n=12 Idh2R140Q male, n=11 Idh2wt female and n=5 Idh2R140Q female)


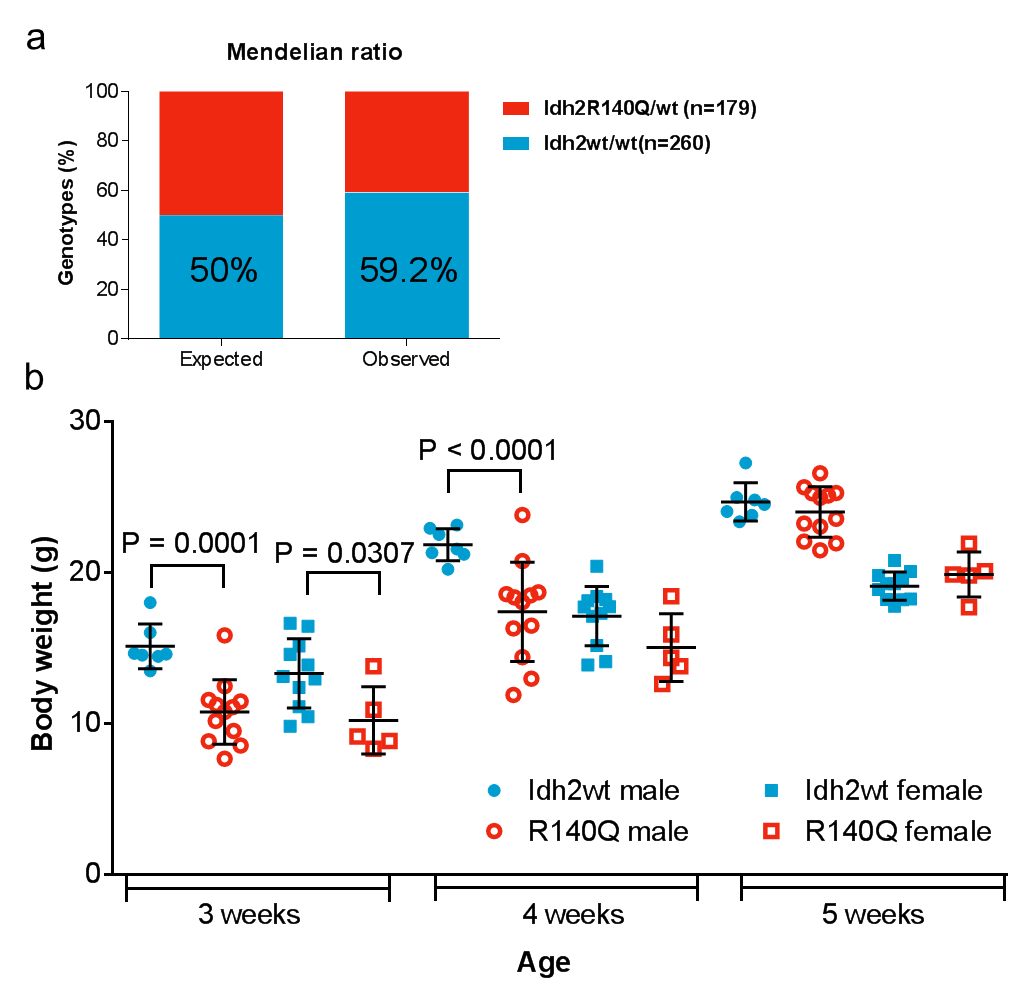

Supplement: Supplementary file 1 — (DOCX 309 kb) [file 10545_2016_9960_MOESM1_ESM.docx]
